# Supplementary material for: Mitochondrial origins of fractional control in regulated cell death
Source: Nat Commun. 2019 Mar 21;10:1313. doi: 10.1038/s41467-019-09275-x (PMC6428895; doi:10.1038/s41467-019-09275-x)
Supplement: Supplementary file 1 — Supplementary Information [file 41467_2019_9275_MOESM1_ESM.pdf]

Supplementary Information

# Mitochondrial origins of fractional control in regulated cell death

Santos *et al.*

## Supplementary Note 1 Flow cytometry gating strategy

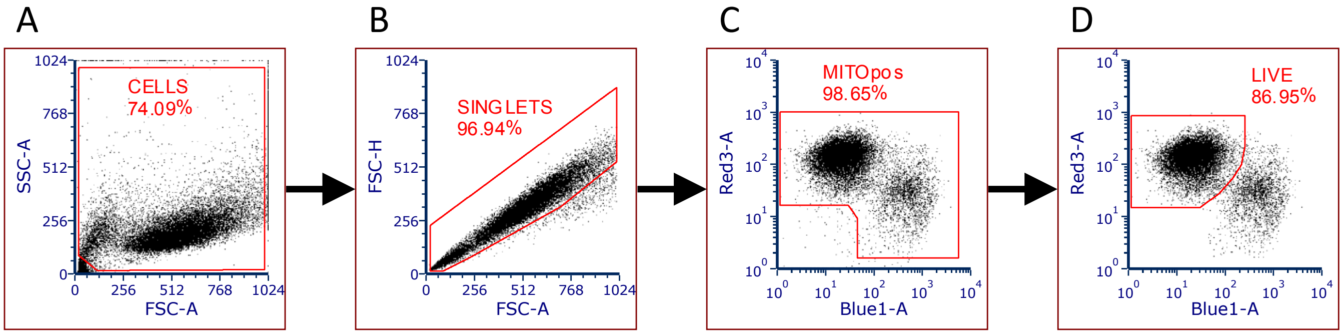


Supplementary Figure 1: **Flow cytometry gating strategy, related to Figure 1 of the main text.** (A) Elimination of debris measurements identified as FSC-A low and SSC-A low. (B) Elimination of doublet measurements by gating along the region FSC-A = FSC-H. (C) Gate on cells that were successfully stained by Mitotracker Deep Red. (D) Live cells identified as Annexin V negative.

# Supplementary Note 2 Mitochondria dynamics

Mitochondria are constantly produced and degraded in cells. As with many other biological components production and degradation processes are fundamentally stochastic, and in result the abundances of mitochondria per cell change in time. To check whether mitochondria abundances change in the four hour experiments presented in the main text, we measured the average and the temporal correlation function of mitochondria dynamics by flow cytometry.

We first measured the average degradation dynamics of mitochondria. Jurkat cells were labeled with Mitotracker Green at time point 0. Then two sets of samples were collected at time points 0, 2, 6 and 25 hours. The first set, designated the experimental group, were immediately prepared for flow cytometry measurements. While the second set, the control group, were relabeled with Mitotracker Green. The control group allowed us to have a time dependent calibration tool, as the average mitochondria abundance is assumed to be constant in time.

Supplementary Figure 2A shows the degradation dynamics of the Mitotracker Green signal. Here we see that for the first six hours the average abundance of mitochondria remains constant. However, after 25 hours, we see that only approximately 50% of the original mitochondria remain. Moreover, in Supplementary Figure 2B we see that the entire probability density of single cell mitochondria abundances behaves similarly.

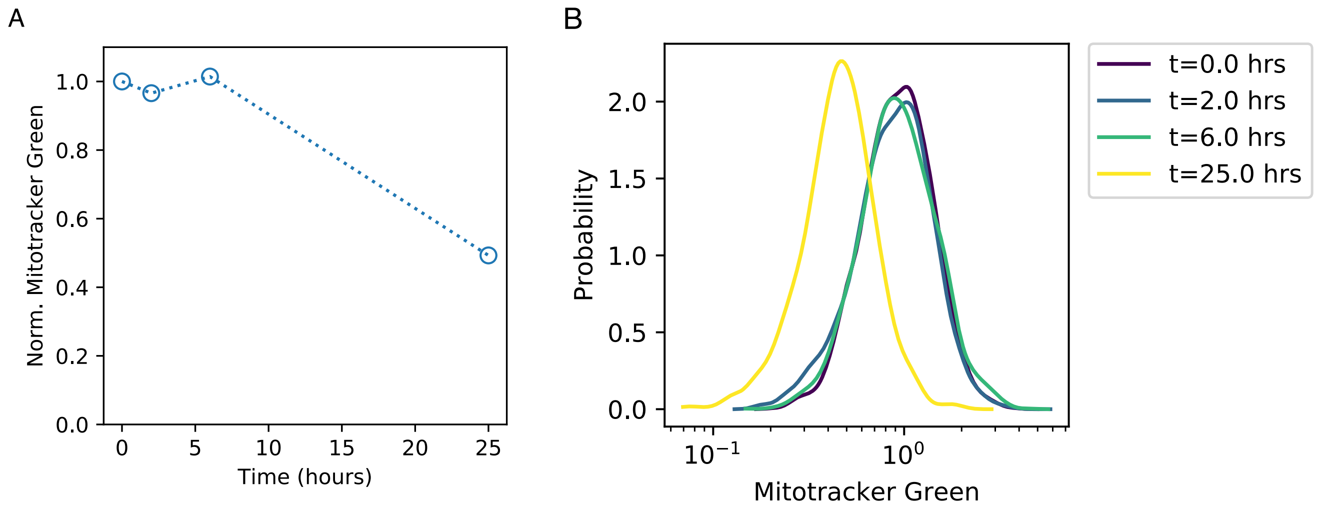


Supplementary Figure 2: **Mitochondria decay dynamics**.

As a secondary evaluation of mitochondria dynamics we probed the correlation of single cell mitochondria abundance across time. The basis of the experiment is to determine the time-scale of the stochastic dynamics of mitochondria production and degradation. Indeed, if the production of mitochondria exhibits large and frequent fluctuations, then the amount of mitochondria measured at two different times will be unique. This uniqueness of the number of mitochondria per cell can be quantified by computing the correlation of mitochondria between two time points.

In an ideal setting, we would measure the amount of mitochondria in individual cells across time. From which we would compute the temporal correlation function. Instead, we assume that the mitochondria dynamics are ergodic, that is the temporal statistics of a single cell is the same as the statistics of a group of cells evaluated at a single time point. We apply the ergodic assumption by administering two spectrally distinct fluorescent labels, $l_1$ and $l_2$, of mitochondria at unique times to a single population of cells. In such an experiment the first mitochondria label will represent the amount of mitochondria per cell at $t_0$. Then the next label applied at $t_1 = t_0 + \tau$ will measure all the mitochondria there were present at $t_0$ and any new mitochondria produced in the time $\tau$. The correlation of mitochondria between these two time points is simply the correlation of $l_1$ and $l_2$ among single cells.

Supplementary Figure 3A presents the experimental design, in which we prepare five unique samples that are representative of the correlation in mitochondria at five different time points. All the samples were stained with Mitotracker Green at time point 0, followed by a staining with Mitotracker DeepRed at a sample dependent time point. The amount of time ($\tau$) between the initial Mitotracker Green and Mitotracker DeepRed labeling is representative of the correlation of mitochondria abundance in single cells at times $t_i$ and $t_j = t_i + \tau$.

Supplementary Figure 3B shows the resulting correlation in time series $\tau$ =0, 2, 4, 11 and 24 hours. Here we see that samples are highly correlated at the initial time points, and then the correlation decays after ≈10 hours. This result suggests that the mitochondria labeled at time zero is a good estimate of the single cell abundance of mitochondria after four hours, as implemented in our results presented in the main text.

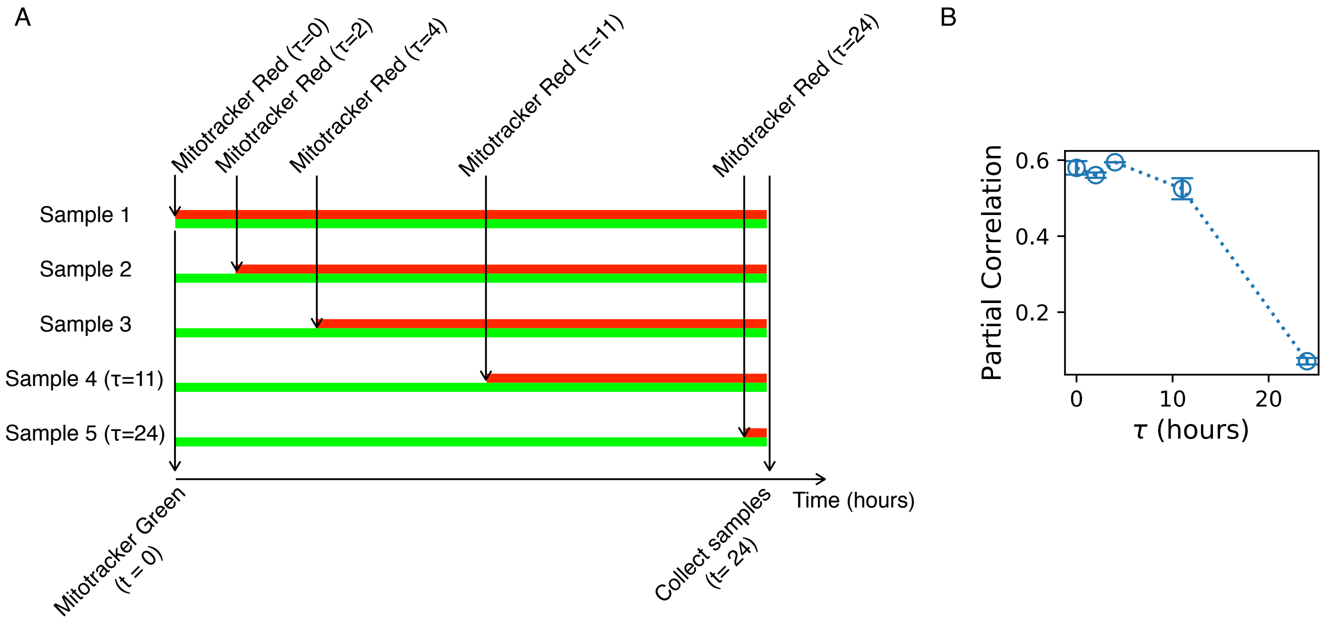


Supplementary Figure 3: **Correlation function of mitochondria dynamics.** (A) The experimental design for measuring the temporal correlation function of mitochondria abundance in single Jurkat cells. (B) The measured partial correlation between Mitotracker DeepRed and Mitotracker Green, where data points and error bars representative of the mean and S.E.M. of experimental duplicates.

The correlation presented in Supplementary Figure 3B is the partial correlation between Mitotracker DeepRed and Mitotracker Green stain. This was necessary on account that each stain is correlated with cell size (Supplementary Figure 4A, B). Eliminating the contribution of cell size to the correlation is important because it allows us to distinguish between two likely scenarios.

The first is displayed in Figure 4D, here the measured correlation between Mitotracker Deep Red and Green is due to their independent correlations with cell size. This would indicate that either the fluctuations in mitochondria were so fast that we could not resolve the correlation function or a problem with our experimental design. Under such a scenario we would measure zero partial correlation between Mitotracker stains for all time points $\tau$, which is not observed in Supplementary Figure 3B.

The second scenario, displayed in Supplementary Figure 4E, is that all the components are correlated. If in this setting we eliminate the contribution of FSC levels in the single cell measurements of mitochondria, we would still measure a nonzero correlation between the the fluorescence of mitochondria stains. Indeed, this is what we observe in Supplementary Figure 3B.

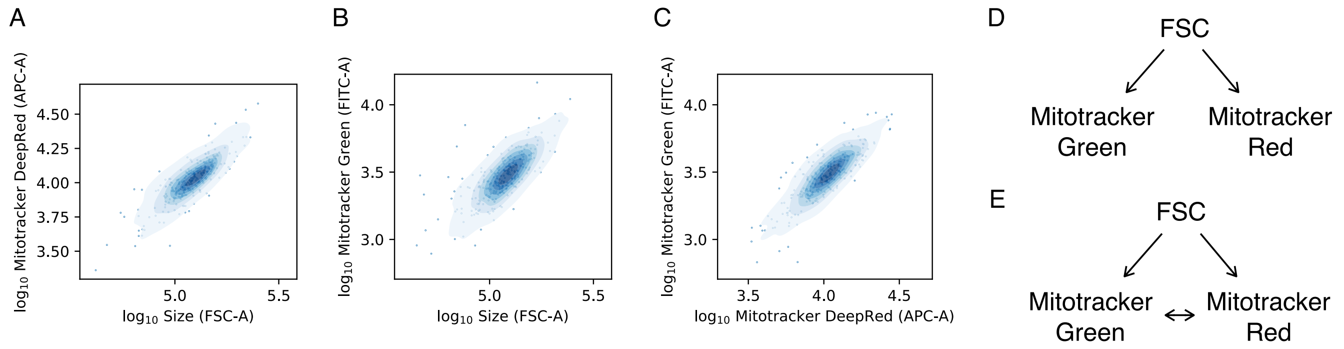


Supplementary Figure 4: **Elimination of cell size influence in correlations.** (A-C) The single Jurkat cell sizes and mitochondria abundance as measured by Mitotracker DeepRed and Mitotracker green for $\tau = 0$. The observed correlation in (C) is attributable to either model (D) or model (E). By applying Partial correlation analysis we are able to distinguish between each model.

# Supplementary Note 3 The DEPICTIVE methodology

In this section we derive our method for DEtermining Parameter Influence on Cell-to-cell variability Through the Inference of Variance Explained, DEPICTIVE.

## Supplementary Note 3.1 Statistics of single cell sensitivities

Consider a population of cells treated with a single dose of a ligand (L) that induces a binary biological response. For example, the administration of the ligand TRAIL induces cells to transition from a live to a dead state. After a sufficient amount of time for the response to manifest, we record the state of each cell $i$ as a positive ($c_i = 1$) or negative ($c_i = 0$) state. The specific encoding of the binary state is not important so long as it remains consistent throughout the analysis. Let $s = \log([\mathrm{L}])$, and $\kappa_i$ be the value of $s$ that causes the $i^{th}$ cell to transition from $c_i = 1 \rightarrow 0$. Then, for any given dose of the stimulus ($s$) the cells that transition from the positive to the null states are those where $\kappa \leq s$. In consequence, we can compute the fraction of cells that remain in the positive state given $s$ from the cumulative distribution of single cell sensitivities, $\kappa$,

$$P(\text{alive}|s) = \int_{\kappa > s} P(\kappa) d\kappa = 1 - P(\kappa \leq s).$$

Traditionally, the binary response of a population to a stimulus is measured by the empirical fraction of cells that respond when treated with distinct doses. This is the so-called dose response curve, and it often takes a sigmoidal shape that is well approximated by the parametric Hill function. The Hill function has explicit parameters that represent the dose $s$ in which 50% of the population responds (e.g. $\mathrm{IC}_{50}$), the fraction of cells sensitive to the stimuli (amplitude and background), and the steepness of the sigmoid (Hill coefficient $n$). As such, the Hill model represents the parametric form of the cumulative distribution of single cell sensitivities,

$$1 - P(\kappa \leq s_l) = \frac{1}{1 + e^{n(s-\mu)}},$$

where $\mu = \log(\mathrm{IC}_{50})$, the amplitude is 1, and the background is 0. Note, that without loss of generality we model a response in which the fraction of cells in a given state depreciates with increased stimulus dose. From the definition of the cumulative distribution we compute the probability density of single-cell sensitivities to $s$ ($\kappa$) by differentiation,

$$P(\kappa) = \frac{n}{4} \frac{1}{\cosh^2\left(\frac{n}{2}(\kappa - \mu)\right)}. \tag{1}$$

The correspondence between the statistics of $\kappa$ and the Hill function parameters comes naturally from Eq. 1. The first moment is simply

$$\langle \kappa \rangle = \mu = \log(\mathrm{IC}_{50}), \tag{2}$$

a fact that is simply determined by the symmetry of the inverse squared hyperbolic cosine. The variance of $\kappa$,

$$\left\langle (\kappa - \mu)^2 \right\rangle = \frac{n}{4} \int_{-\infty}^{\infty} \frac{(\kappa - \mu)^2}{\cosh^2\left(\frac{n}{2}(\kappa - \mu)\right)} d\kappa$$

is a bit more challenging. Consider the change of variable $x = \frac{n}{2}(\kappa - \mu)$

$$P(x) = \int_0^{\infty} \frac{x^2}{\cosh^2(x)} dx,$$

and recall that

$$\int_{x=0}^{\infty} \frac{x^k}{\cosh^2(x)} dx = 2^{1-k}\left(1 - 2^{1-k}\right)\Gamma(k+1)\zeta(k),$$

valid for $k \neq 1$ and $\zeta$ is the Riemann zeta function. Incorporating $k = 2$ we find that,

$$\left\langle (\kappa - \mu)^2 \right\rangle = \frac{\pi^2}{3} \frac{1}{n^2}. \tag{3}$$

Overall, we see that the $\mathrm{IC}_{50}$ and the Hill coefficient are related to the mean (Eq. 2) and the variance (Eq. 3) of single cell sensitivities to the stimulus, respectively.

## Supplementary Note 3.2 Decomposing sources of variability

Consider a cell composed of $N$ biological components and their corresponding abundances encoded by $\mathbf{x} = [x_1, x_2, \ldots, x_N]^T$. Together, $\mathbf{x}$ determines the cell's sensitivity ($e^\kappa$) to TRAIL by some unknown function $g(\mathbf{x})$. We do not have *a priori* knowledge of this function, however let's temporarily assume that we can compute its Taylor expansion about $\langle \mathbf{x} \rangle$. The expansion of $\log(g(\mathbf{x})) = \kappa$ is then,

$$\kappa = \log(g(\langle \mathbf{x} \rangle)) + \sum_{i=1}^{N} \left. \frac{\partial \log(g)}{\partial x_i} \right|_{x_i = \langle x_i \rangle} \delta x_i + \ldots \tag{4}$$

where $\delta x_i = x_i - \langle x_i \rangle$. In Eq. 4 the logarithm of single cell sensitivities are related to the linear CCV of the biological constituents. However, from our data we observe that CCV are more appropriately described in logarithmic scale. If we examine the expansion of the log abundances of the biological constituents,

$$\log(x_i) = \log(\langle x_i \rangle) + \frac{\delta x_i}{\langle x_i \rangle} - \frac{\delta x_i^2}{2 \langle x_i \rangle^2} + \cdots .$$

and assume that the CCV is smaller than the mean [12], we may approximate the logarithm to first order as,

$$\delta \log(x) = \log(x) - \log(\langle x \rangle) \approx \frac{\delta x}{\langle x \rangle}.$$

Incorporating this approximation into Eq. 4,

$$\kappa \approx \log(g(\langle \mathbf{x} \rangle)) + \sum_{i=1}^{N} \left. \frac{\partial \log(g)}{\partial \log(x_i)} \right|_{x_i = \langle x_i \rangle} \delta \log(x_i). \tag{5}$$

Note that we have expressed the derivative with respect to $\log(x_i)$, by application of the chain rule, $\partial_{x_i} f = \frac{1}{x_i} \partial_{\log(x_i)} f$ with $f$ being an arbitrary differentiable function of $x_i$.

Using Eq. 5 we compute the statistics of $\kappa$ as they depend on the CCV of each biological component. Applying Eq. 2,

$$\langle \kappa \rangle = \log(\mathrm{IC}_{50}) \qquad \Longrightarrow \qquad \log(\mathrm{IC}_{50}) = \log(g(\langle \mathbf{x} \rangle)).$$

In similar fashion, we compute the second central moment

$$\left\langle (\kappa - \log(\mathrm{IC}_{50}))^2 \right\rangle = \left\langle \left( \sum_{i=1}^{N} k_i \delta \log(x_i) \right)^2 \right\rangle$$

where $k_i = \partial_{\log(x_i)} \log(g)|_{x_i = \langle x_i \rangle}$. Indeed, the coefficients $k_i$ are not random variables, resulting

$$\left\langle (\kappa - \log(\mathrm{IC}_{50}))^2 \right\rangle = \mathbf{k}^T \mathbf{C} \mathbf{k} \tag{6}$$

where $\mathbf{C}$ is the covariance matrix of the the log abundances of each component in the biological system. When the logarithm of the abundances of the biological components are not correlated, $\mathbf{C}$ is diagonal. Consequently Eq. 6 shows that the variance of single-cell sensitivities to ligand emerging from CCV is simply a weighted sum over the variances of each biological component with weights equal to the coefficients $k_i^2$.

The expansion to first order (main text Eq. 1) and the correspondence between the Hill model parameters and the statistics of $\kappa$ (Equations 2, 3) represent the center piece of the DEPICTIVE methodology.

### Supplementary Note 3.2.1 Variance explained

Our interest is in quantifying the extent to which the CCV of specific components contribute to the CCV in sensitivities to a stimulus. Ideally, this would be determined experimentally, where cells are engineered such that there is no CCV in component $j$ while all other components are unperturbed. In such an experiment the variance of single cell sensitivities is attributed to the CCV of all components that are not $j$,

$$\left\langle \delta \kappa^2 | \delta \log(x_j) \right\rangle = \sum_{i \neq j} k_i^2 C_{ii}. \tag{7}$$

Consequently, the CCV attributable to $j^{th}$ component is the difference between the estimated variance of single cell sensitivities between the non-engineered (Eq. 6) and engineered cells (Eq. 7),

$$\left\langle \delta\kappa^2 \right\rangle - \left\langle \delta\kappa^2 | \delta \log(x_j) \right\rangle = k_j^2 C_{jj}. \tag{8}$$

Lastly, we can quantify the relative contribution of the $j^{th}$ component to the observed CCV by taking the ratio of variances,

$$\text{Variance Explained} = \frac{\text{Variance attributed to } j}{\text{Variance of non-engineered cells}} = \frac{\left\langle \delta\kappa^2 \right\rangle - \left\langle \delta\kappa^2 | \delta \log(x_j) \right\rangle}{\langle \delta\kappa^2 \rangle}$$

We can re-express the variance explained in terms of the available information by substituting Eq. 8 for the variance attributed to $j$ and using Eq. 3 for the variance of the non-engineered cells,

$$\text{Variance Explained} = k_j^2 C_{jj} \frac{3n^2}{\pi^2}. \tag{9}$$

Importantly, the variance $C_{jj}$ is that of the non-engineered cells, and not the engineered cells where $C_{jj}^{\text{engineered}} = 0$. Eq. 9 shows us that we can estimate the contribution of any component $j$ to the observed diversity of single sensitivities without needing to measure every component in the biological system being studied.

In practice, experimentally eliminating the CCV of one component of a network at a time is infeasible. This is on account that experimental tools for producing a population of cells with exactly $x_j$ number of molecules $j$ do not exists, and may actually be fundamentally impossible to construct [4,6,7]. However, the expression we derived from this hypothetical experiment is useful when combined with single cell data. Indeed, single cell measurements provide a means for estimating the covariance matrix of cellular components $\mathbf{C}$ and when combined with a dose response experiment $n$ can be inferred. In the next sections we develop a strategy for estimating the last unknown parameter in Eq. 9, $k_j$.

## Supplementary Note 3.3 Inferring phenomenological parameters

In this section we introduce the single cell Hill model and how to infer its parameters from single cell dose response data. Application of our model and inference of its parameters provide the necessary information for estimating the variance explained introduced in Eq. 9.

### Supplementary Note 3.3.1 The single cell Hill model

Recall the Hill model applied to dose response data where the response depreciates with the ligand dose ($L$),

$$f(L; \text{IC}_{50}, n) = \frac{1}{1 + \left(\frac{L}{\text{IC}_{50}}\right)^n} = \left[1 + e^{n(\log(L) - \log(\text{IC}_{50}))}\right]^{-1}. \tag{10}$$

We then incorporate measured components by substituting $n$ with $n_{\mathbf{x}}$ and $\log(\text{IC}_{50})$ with $\log(\text{IC}_{50}(\delta \log(\mathbf{x})))$. First, recall that the Hill coefficient is related to the diversity of single cell sensitivities originating from unmeasured components. Then by using Eq. 3 and Eq. 6

$$n_{\mathbf{x}}^2 = \frac{\pi^2}{3 \left\langle \delta\kappa^2 | \delta \log(\mathbf{x}) \right\rangle} = \frac{\pi^2}{3(\langle \delta\kappa^2 \rangle - \mathbf{k}_{\mathbf{x}}^T \mathbf{C}_{\mathbf{x}} \mathbf{k}_{\mathbf{x}})} \tag{11}$$

where $\mathbf{k}_{\mathbf{x}}$ and $\mathbf{C}_{\mathbf{x}}$ is the column vector of coefficients and the covariance matrix of the log abundances from single cell measurements of each measured component, respectively. Next, recall from Eq. 2 that the average sensitivity is simply the $\log(\text{IC}_{50})$. Then, given measurements $\delta \log(\mathbf{x})$,

$$\langle \kappa | \delta \log(\mathbf{x}) \rangle = \log(\text{IC}_{50}(\delta \log(\mathbf{x}))) = \log(\text{IC}_{50}) + \mathbf{k}_{\mathbf{x}}^T \delta \log(\mathbf{x}) \tag{12}$$

Then by substituting Eqs. 11, 12 into Eq. 10 results in the single cell Hill model,

$$f(L; \text{IC}_{50}, n_{\mathbf{x}}, \mathbf{k}, \delta \log(\mathbf{x})) = \left[1 + e^{n_{\mathbf{x}}(\log(L/\text{IC}_{50}) - \mathbf{k}^T \delta \log(\mathbf{x}))}\right]^{-1}. \tag{13}$$

### Supplementary Note 3.3.2 A framework for fitting

Application of our first order theory to single cell data amounts to inferring the parameters of the population dose response (amplitude $A$, background $b$, $\text{IC}_{50}$, $n$) and the coefficients of measured components $\mathbf{k_x}$. Indeed, the amplitude and background are important parameters in practice as it is often the case that cell cultures for zero and effectively infinite ligand concentration contain cells in both positive and null states. For example, in some cases cultures with zero TRAIL exhibit only ≈80% and with maximum TRAIL ≈10% of cells in the live state. Applying the complete set of parameters the single cell Hill of Eq. 13 model becomes,

$$f(L; \text{IC}_{50}, n_{\mathbf{x}}, \mathbf{k}, \delta\log(\mathbf{x})) = A\left[1 + e^{n_{\mathbf{x}}(\log(L/\text{IC}_{50}) - \mathbf{k}^T\delta\log(\mathbf{x}))}\right]^{-1} + b \tag{14}$$

We use a piece-wise strategy to infer the parameters from Eq. 14. We assume that for each dose of ligand $L$ we have a table containing the abundances of biological constituents and cell state for each cell in the sample.

First, we are interested in the fractional response of cell's to the ligand, this is captured by the fraction of cells alive for each dose of ligand. Empirically, we estimate this quantity for dose $L$ as

$$\hat{p}_L = \frac{\hat{M}_{L,\text{alive}}}{\hat{M}_L} \tag{15}$$

where $M_{L,\text{alive}}$ and $M_L$ are the number of live and total number of cells measured at dose $L$, respectively. Now, in order to infer the single cell Hill model parameters from $\hat{p}_L$ consider the definition of the probability of a cell being alive given $L$,

$$P(\text{alive}|L) = \int_{\mathbf{y}} P(\text{alive}, \mathbf{y}|L)d\mathbf{y} \tag{16}$$

where for simplicity we made the substitution $\mathbf{y} = \delta\log(\mathbf{x})$. We don't know the joint distribution $P(\text{alive}, \mathbf{y}|L)$ as written, however we can re-express the joint distribution by the following product

$$P(\text{alive}, \mathbf{y}|L) = P(\text{alive}|\mathbf{y}, L)P(\mathbf{y}|L), \tag{17}$$

in which each term is known. Inspection of Eq. 17 makes this clear. First, the conditional distribution is related to the single cell Hill model (Eq. 14) by $P(\text{alive}|\mathbf{y}, L) = (f(L; \text{IC}_{50}, n_{\mathbf{y}}, \mathbf{k}, \mathbf{y}) - b)/A$. Second, we have an estimate of joint distribution $P(\mathbf{y}|L)$ of biological components from the single cell measurements. Then by substitution of Eq. 17 into Eq. 16 and incorporating our knowledge of $\hat{p}_L$ from Eq. 15 we arrive at our estimate,

$$\hat{p}_L = \frac{1}{\hat{M}_L}\sum_{j=1}^{\hat{M}_L} f(L; \text{IC}_{50}, n_{\mathbf{y}}, \mathbf{k}, \hat{\mathbf{y}}_j) \tag{18}$$

in which we substituted $P(\mathbf{y}|L)$ with the empirical distribution $\frac{1}{\hat{M}_L}\sum_{j=1}^{\hat{M}_L}\delta(\hat{\mathbf{y}}_j - \mathbf{y})$. Note that here $\delta$ is the Dirac delta function, which is defined as

$$\delta(\mathbf{y}_j - \mathbf{y}) = \begin{cases} 0 & \text{if } \mathbf{y} \neq \mathbf{y}_j \\ \infty & \text{if } \mathbf{y} = \mathbf{y}_j \end{cases} \qquad \text{and} \qquad \int_{\mathbf{y}} \delta(\mathbf{y}_j - \mathbf{y})d\mathbf{y} = 1.$$

Eq. 18 shows us how to quantitatively relate our single cell Hill model and its corresponding parameters to the empirical probability that any given cell is alive given a dose $L$.

Secondly, on account that we have knowledge of the each cell's state label we can measure the average of each biological component conditioned that a cell is alive. Our empirical estimate of the conditional average is simply

$$\hat{\boldsymbol{\mu}}_{L,\text{alive}} = \frac{1}{\hat{M}_{L,\text{alive}}}\sum_{\{j|c_j=1,L\}} \hat{\mathbf{y}}_{j,L}. \tag{19}$$

Now to connect this measurement to the single cell Hill model recall the definition of the conditional average,

$$\langle\mathbf{y}|\text{alive}, L\rangle = \int_{\mathbf{y}} \mathbf{y}P(\mathbf{y}|\text{alive}, L)d\mathbf{y}. \tag{20}$$

Again, we are faced with a probability distribution $P(\mathbf{y}|\text{alive}, L)$ which, as written, does not have an explicit functional form. By Bayes' theorem we are able to manipulate $P(\mathbf{y}|\text{alive}, L)$ into a recognizable form,

$$P(\mathbf{y}|\text{alive}, L) = \frac{P(\text{alive}|\mathbf{y}, L)P(\mathbf{y}|L)}{P(\text{alive}|L)}, \tag{21}$$

where as before $P(\text{alive}|\mathbf{y}, L)$ is related to the single cell Hill model, $P(\text{alive}|L)$ can be estimated from the single cell Hill model, and $P(\mathbf{y}|L)$ is simply the joint distribution of molecular components. If we apply the empirical distribution of biological components $P(\mathbf{y}|L)$ and substitute Eq. 21 into Eq. 20 we see that

$$\hat{\mu}_{L,\text{alive}} = \frac{1}{\hat{P}(\text{alive}|L)} \frac{1}{\hat{M}_L} \sum_{j=1}^{\hat{M}_L} \mathbf{y}_j \frac{f(L; \text{IC}_{50}, n_{\mathbf{y}}, \mathbf{k}, \hat{\mathbf{y}}_j) - b}{A}. \tag{22}$$

where $\hat{P}(\text{alive}|L) = \frac{1}{\hat{M}_L} \sum_{j=1}^{\hat{M}_L} \frac{f(L;\text{IC}_{50}, n_{\mathbf{y}}, \mathbf{k}, \hat{\mathbf{y}}_j) - b}{A}$.

Together Eqs. 18, 22 are the equations used in our piecewise fitting strategy. The strategy is as follows:

1. Use nonlinear optimization (SciPy's optimize.fmin [9]) to infer $A, \text{IC}_{50}, n, b$ holding $k_1, k_2, \ldots k_N$ constant by computing the absolute value of the difference between the LHS and RHS of Eq. 18. Return the sum of these differences over each dose $L$. Store the error in a variable $\epsilon_p$.

2. Use nonlinear optimization (SciPy's optimize.fmin [9]) to infer $k_1, k_2, \ldots, k_N$ holding $A, \text{IC}_{50}, n, b$ constant by computing the absolute value of the difference between the LHS and RHS of Eq. 22. Return the sum of these differences over each dose $L$. Store the error in a variable $\epsilon_\mu$.

3. Repeat steps 1 and 2 until $|\epsilon_\mu^{(q)} + \epsilon_p^{(q)} - \epsilon_\mu^{(q-1)} - \epsilon_p^{(q-1)}| < \epsilon_T$, where $q$ is the most recent iteration and we set $\epsilon_T = 10^{-8}$.

### Supplementary Note 3.3.3 Application to Mitotracker data

Each sample collected from a TRAIL dose $j$ consists of $N_j$ cells where each $i$ cell is represented by the tuple $(\rho_i, y_i)$. Here, $\rho_i$ represents the mitochondria density measurement, while $y_i$ is a binary variable representing cell state. The $i^{th}$ cell is endowed the label $y_i = 1$ if it is living (Annexin V negative), and $y_i = 0$ (Annexin V positive) otherwise.

As defined, the parameters of interest can be simply inferred using the protocol described above. However, we cannot employ this standard strategy, because the MitoTracker Deep Red signal depends on the electrochemical properties of the mitochondria. These properties are different for living and dead cells. In consequence, we do not have measurements representing the joint distribution ($P(y|L)$ in the previous section) of biological components for each dose of TRAIL. To circumvent this problem we make a modest assumption that allows us to fit the data.

We assume that TRAIL does not influence the abundance of mitochondria per cell in the four hours the experiment is conducted. Under this assumption we define,

$$P(\log(\rho)|T) = P(\log(\rho)) = P(\log(\rho)|T < \phi), \tag{23}$$

where we take $\phi$ to be the empirical $\text{IC}_{15}$ from each data set and we normalize $P(\log(\rho)|T)$ between 0 and 1. We chose the $\text{IC}_{15}$ due to the variability in mitochondria staining across samples. With this approximate joint distribution we are able to apply our fitting protocol.

### Supplementary Note 3.3.4 Simulations for testing DEPICTIVE

To test our statistical framework and inference strategy we simulated the response of virtual cells to TRAIL. Here, we assume that all the cell-to-cell variability is accounted for by five components, namely $x, w_1, z, q$, and $w_2$. The $j^{th}$ cell is then simulated by

$$x_j \sim \mathcal{N}(0,1), \qquad w_{1j} \sim \mathcal{N}(0,1), \qquad q_j \sim \mathcal{N}(0,1), \qquad z_j \sim \mathcal{N}(0,1), \text{ and } \qquad w_{2j} \sim \mathcal{N}(0,1)$$

and in consequence the tuple $(x_j, w_{1j}, q_j, z_j, w_{2j})$ represent the natural logarithm of abundances of each biological constituent. The corresponding sensitivity to TRAIL is then computed by,

$$\kappa_i = \sum_{i \in \{x, w_1, z, q, w_2\}} k_i i.$$

In Figure 3 of the main text we set $k_x = 0.25$, $k_{w_1} = 0.5$, $k_z = 1$, $k_q = 1.5$, and $k_{w_2} = 2$. The cell state of the $j^{th}$ cell is binary, with $y_j = 1$ representing a live cell and $y_j = 0$ a dead cell. Then the cell state for the $k^{th}$ dose $s_k = \log([\mathrm{TRAIL}]_k/\mathrm{IC}_{50})$ is

$$y_{kj} = \begin{cases} 1 & \text{if } s_k < \kappa_j \text{, and} \\ 0 & \text{if } s_k \geq \kappa_j . \end{cases}$$

To simulate a dose response we logarithmically vary the dose, $s$, in the range $0.00094 \leq s \leq 1060.94093$.

# Supplementary Note 4 Application of inference method to data

In each figure panel we show detailed analysis of our single cell data. Importantly we analyze the density of Mitotracker DeepRed (MDR) stain $\rho$ and side scatter (SSC/FSC) from live cells. We applied the DEPICTIVE analysis to the side scatter density so that each experiment has a negative control. Indeed, we do not expect that the CCV in SSC/FSC to explain the diversity in single cell sensitivities to TRAIL. Together, the DEPICTIVE strategy allows for transparent decomposition of sources of CCV from single cell data.

## Supplementary Note 4.1 Analysis of Jurkat Cells

Table 1: **Variance explained.** The variance of single cell sensitivities explained by $\rho$ and SSC/FSC in Jurkat cells.

| Experiment | Variance Explained ($\rho$) | Variance Explained (SSC/FSC) |
|---|---|---|
| Jurkat 1, Supplementary Figure 5 | 0.325 | 0.001 |
| Jurkat 2, Supplementary Figure 6 | 0.371 | 0.010 |
| Jurkat 3, Supplementary Figure 7 | 0.300 | 0.025 |

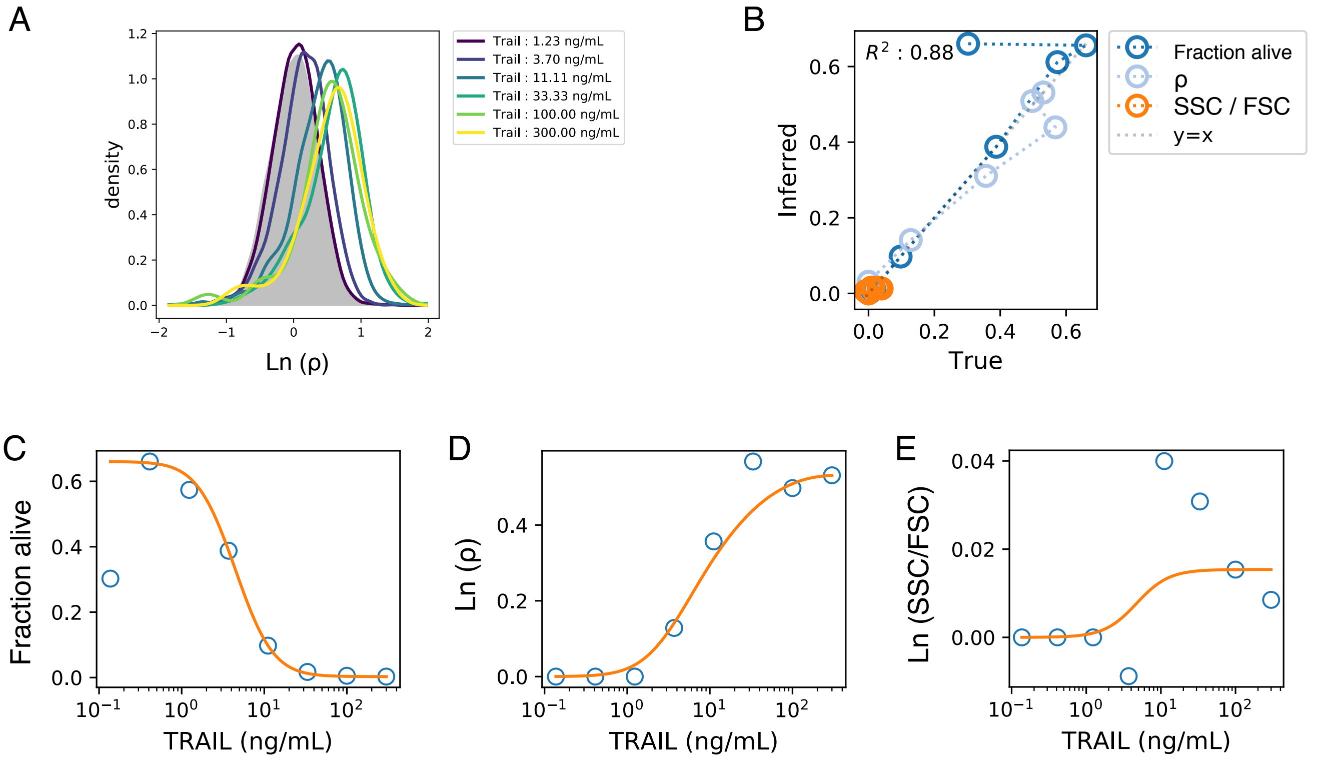


Supplementary Figure 5: **Jurkat, replicate 1 data analysis, related to Figure 4 of the main text.** (A) The distribution of Mitotracker DeepRed density among living cells, $P(\text{Ln}(\rho)|\text{alive}, T)$. (B) The agreement of the inferred and empirical fraction of cells alive, the average mitochondria and SSC/FSC densities of live cells. The measurements and model fits of the (C) fraction of cells alive, (D) the average mitochondria density of live cells, and (E) the average side scatter density estimated by the single cell Hill model using the inferred parameters.

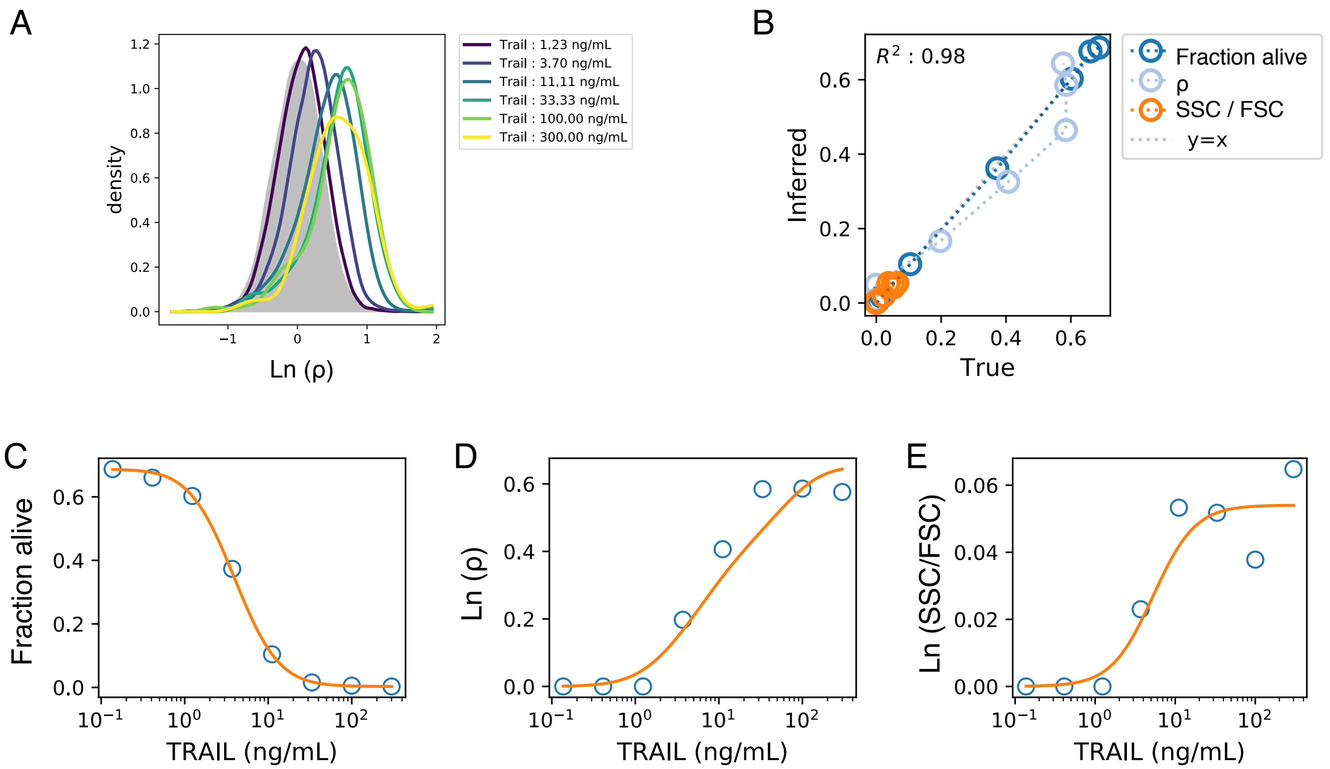


Supplementary Figure 6: **Jurkat, replicate 2 data analysis, related to Figure 4 of the main text.** (A) The distribution of Mitotracker DeepRed density among living cells, $P(\text{Ln}(\rho)|\text{alive}, T)$. (B) The agreement of the inferred and empirical fraction of cells alive, the average mitochondria and SSC/FSC densities of live cells. The measurements and model fits of the (C) fraction of cells alive, (D) the average mitochondria density of live cells, and (E) the average side scatter density estimated by the single cell Hill model using the inferred parameters.

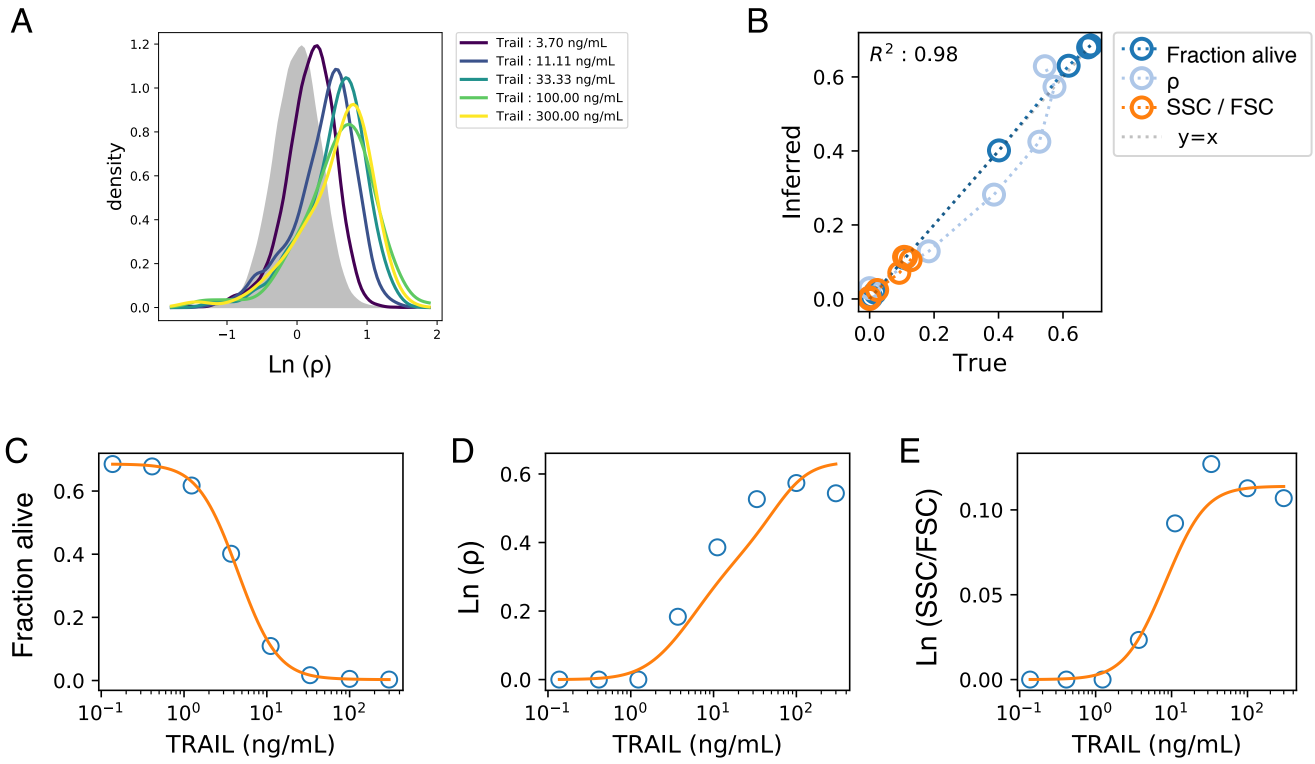


Supplementary Figure 7: **Jurkat, replicate 3 data analysis, related to Figure 4 of the main text.** (A) The distribution of Mitotracker DeepRed density among living cells, $P(\mathrm{Ln}(\rho)|\mathrm{alive}, T)$. (B) The agreement of the inferred and empirical fraction of cells alive, the average mitochondria and SSC/FSC densities of live cells. The measurements and model fits of the (C) fraction of cells alive, (D) the average mitochondria density of live cells, and (E) the average side scatter density estimated by the single cell Hill model using the inferred parameters.

## Supplementary Note 4.2 Analysis of MDA-MB-231 Cells

Table 2: **Variance explained** The variance of single cell sensitivities explained by $\rho$ and SSC/FSC in MDA-MB-231 cells displayed in Supplementary Figure 8.

| [ABT263] ($\mu M$) | Variance Explained ($\rho$) | Variance Explained (SSC/FSC) |
|---|---|---|
| 0 | 0.06710 | 0.01015 |
| 1 | 0.26105 | 0.02254 |
| 10 | 0.09692 | 0.06880 |

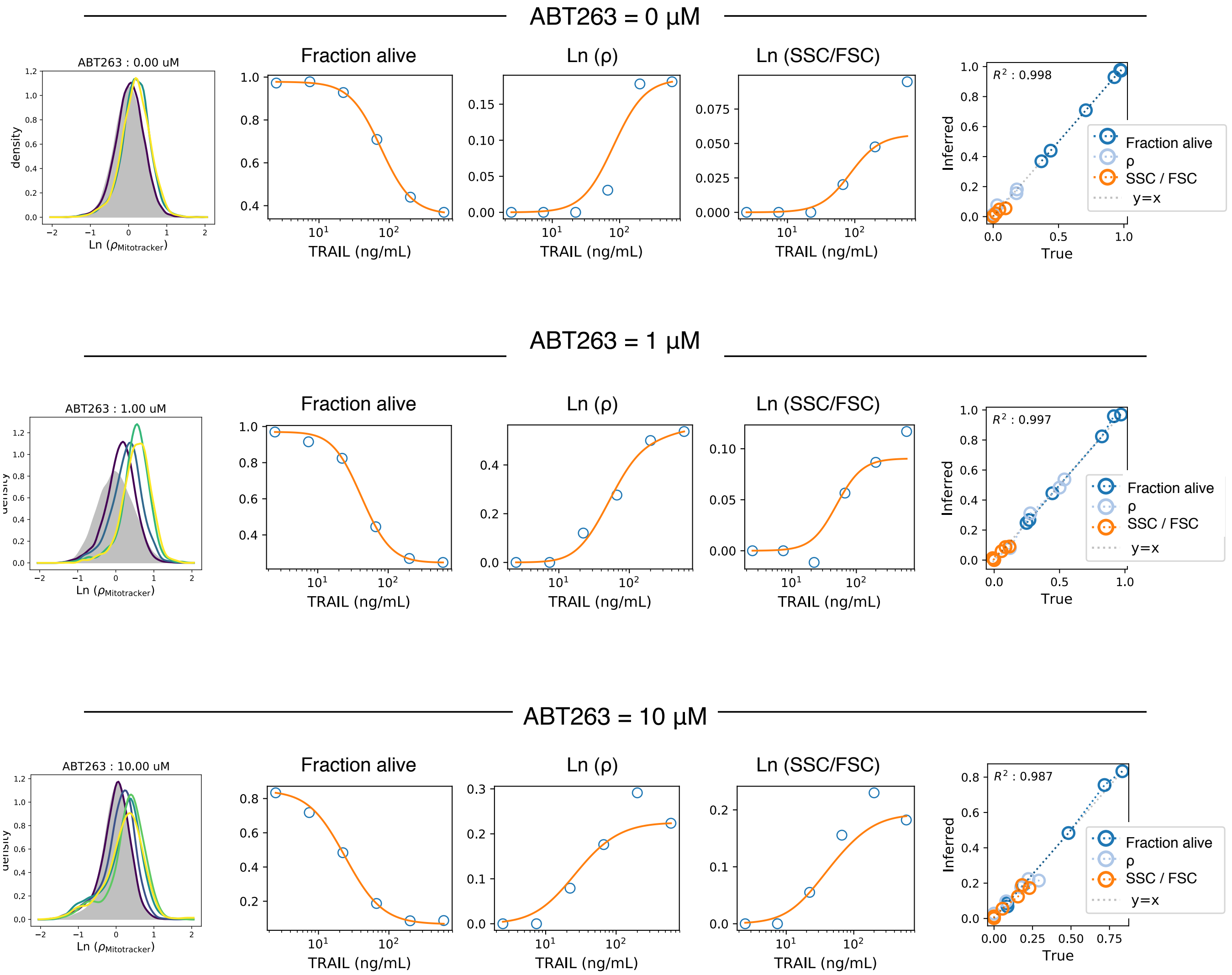


Supplementary Figure 8: **MDA-MB-231, replicate 1 data analysis, related to Figure 4, 6 of the main text.** Each row displays the identical analysis applied to TRAIL dose response data in which a unique dose of ABT263 has been administered to the cell cultures. The analysis of each row, from left to right, consists of the following. The distribution of Mitotracker DeepRed density among living cells, $P(\text{Ln}(\rho)|\text{alive}, T, ABT263)$. The measurements and model fits of the fraction of cells alive, the average mitochondria density of live cells, and the average side scatter density estimated by the single cell Hill model using the inferred parameters. Lastly, the agreement of the inferred and empirical fraction of cells alive, the average mitochondria and SSC/FSC densities of live cells.

Table 3: **Variance explained** The variance of single cell sensitivities explained by $\rho$ and SSC/FSC in MDA-MB-231 cells displayed in Supplementary Figure 9.

| [ABT263] ($\mu M$) | Variance Explained ($\rho$) | Variance Explained (SSC/FSC) |
|---|---|---|
| 0 | 0.00020 | 0.00064 |
| 1 | 0.10715 | 0.00962 |
| 10 | 0.09254 | 0.033947 |
| 20 | 0.02933 | 0.011905 |

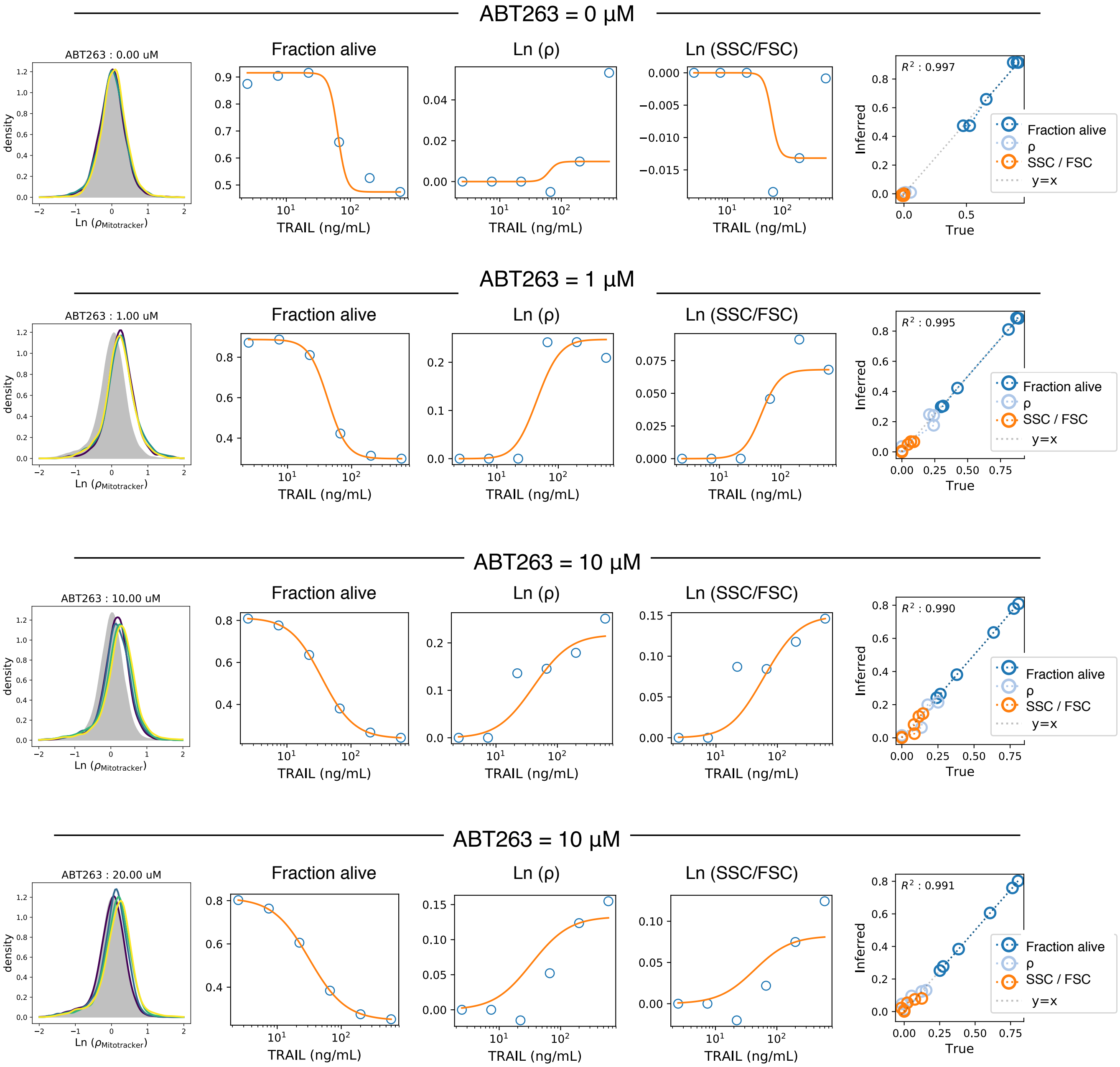


Supplementary Figure 9: **MDA-MB-231, replicate 2 data analysis, related to Figure 4, 6 of the main text.** Each row displays the identical analysis applied to TRAIL dose response data in which a unique dose of ABT263 has been administered to the cell cultures. The analysis of each row, from left to right, consists of the following. The distribution of Mitotracker DeepRed density among living cells, $P(\mathrm{Ln}(\rho)|\mathrm{alive}, T, ABT263)$. The measurements and model fits of the fraction of cells alive, the average mitochondria density of live cells, and the average side scatter density estimated by the single cell Hill model using the inferred parameters. Lastly, the agreement of the inferred and empirical fraction of cells alive, the average mitochondria and SSC/FSC densities of live cells.

Table 4: **Variance explained** The variance of single cell sensitivities explained by $\rho$ and SSC/FSC in MDA-MB-231 cells displayed in Supplementary Figure 10.

| [ABT263] ($\mu M$) | Variance Explained ($\rho$) | Variance Explained (SSC/FSC) |
|---|---|---|
| 0 | 0.037 | 0.030 |
| 1 | 0.080 | 0.026 |
| 3 | 0.092 | 0.106 |
| 10 | 0.068 | 0.077 |

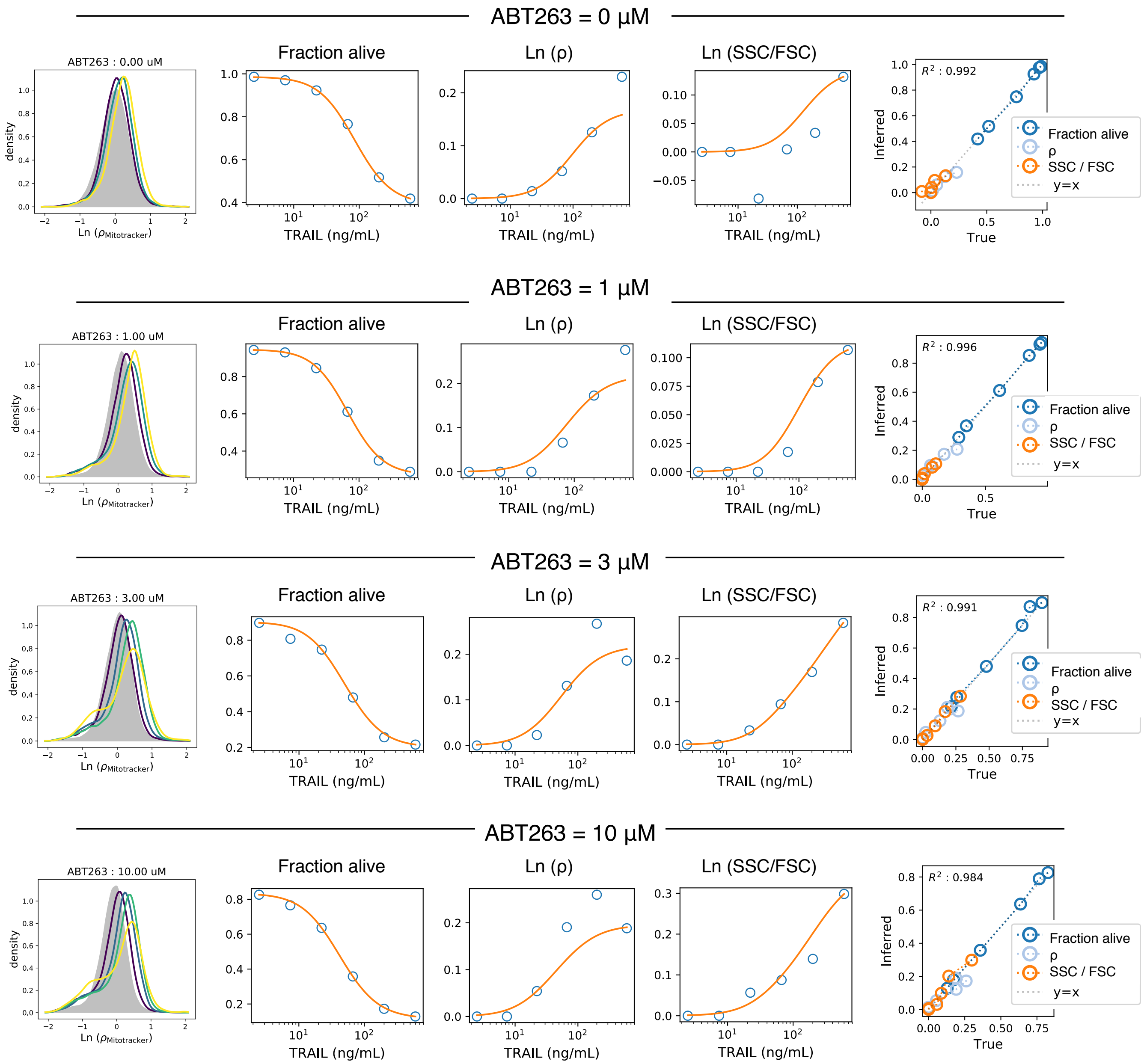


Supplementary Figure 10: **MDA-MB-231, replicate 3 data analysis, related to Figure 4, 6 of the main text.** Each row displays the identical analysis applied to TRAIL dose response data in which a unique dose of ABT263 has been administered to the cell cultures. The analysis of each row, from left to right, consists of the following. The distribution of Mitotracker DeepRed density among living cells, $P(\text{Ln}(\rho)|\text{alive}, T, ABT263)$. The measurements and model fits of the fraction of cells alive, the average mitochondria density of live cells, and the average side scatter density estimated by the single cell Hill model using the inferred parameters. Lastly, the agreement of the inferred and empirical fraction of cells alive, the average mitochondria and SSC/FSC densities of live cells.

# Supplementary Note 5 Single cell measurements in HeLa cells

To test whether we are able to reproduce the observed correlation between single cell sensitivity to TRAIL and mitochondria abundance first documented by Márquez-Jurado *et al.* [11], we performed a TRAIL dose response in HeLa cells. Consistent with the previously published results, the dose response of TRAIL in Figure 11 shows that ≈40% of the cells transition from a live to dead state with very high doses of TRAIL. This is in stark contrast to our observations in Jurkat and MDA-MB-231 cells, in which nearly all cells are sensitive to TRAIL (Supplementary Figures 5, 6, 7), or are coerced to be sensitive with the pan-Bcl-2 inhibitor ABT263 (Supplementary Figures 8, 9, 10), respectively. These results suggest that the parameterization of the extrinsic apoptosis pathway in HeLa are distinct from that in Jurkat and MDA-MB-231 cell lines.

Next, we measured the probability density of single cell mitochondria density ($\rho$) of live cells for different doses of TRAIL. Qualitative analysis of the density plots in Supplementary Figure 11, shows a weak enrichment for cells with relatively high mitochondria densities. We quantified this effect by applying DEPICTIVE to TRAIL dose responses for three distinct doses of ABT263. Supplementary Figure 11 shows that DEPICTIVE fits the data well ($R^2 \geq 0.99$), indicating that the estimates of the variance explained are not incorrect on account of a discrepancy between the model's behavior and the empirical results. In Table 5 we see that the maximum fraction of variance explained by mitochondria density is $\approx 3\%$. This is in distinct contrast to the results of MDA-MB-231 in the main text Figure 6, where the addition of ABT263 increases the variance explained by mitochondria density to ≥25%. Together, our results suggest that mitochondria weakly contributes to the observed diversity of single cell sensitivities to TRAIL.

The data presented in Supplementary Figure 11 establishes that the mitochondria dependent response of HeLa cells to TRAIL, or the combination of TRAIL and ABT263, is fundamentally different from that of Jurkat and MDA-MB-231. We interpret this result to indicate that mitochondria density does not control the propensity of HeLa cells for TRAIL induced apoptosis. Indeed, such a result seems paradoxical to those of Márquez-Jurado *et al.* [11], until we consider the differences in our experimental design and analysis.

In the novel work of Márquez-Jurado *et al.* [11], they measure the time to death of single cells by microscopy. In this experimental assay, they were able to use single cell tracking to link a cell's mitochondria abundance at time 0 with its fate at 24 hours. With this experimental design they were able measure the mitochondria abundances for cells that remained alive and those that transitioned to the dead state. The apparent 5-fold higher $IC_{50}$ we report has to do with the much shorter 4 hour time-span of our experiment and that we computed the effective TRAIL $IC_{50}$ by using the maximum amplitude of the cell death response to TRAIL, which we believe reflects the effective outcome of TRAIL signaling. Indeed, when we calculate relative TRAIL $IC_{50}$ for Hela cells as done by Márquez-Jurado *et al.*, we obtain a much closer $IC_{50}$ value around 80ng/ml. Also, in Figure 2 of their manuscript they show that the average mitochondria abundance for cells that transition from live to dead decreases with TRAIL. Moreover, by qualitative inspection the mean mitochondria abundance among the live cells remains relatively constant. Indeed, as TRAIL increases the average mitochondria abundance among live and dead cells converges, which is consistent with their quantitative analysis by measuring the AUC. While a truly interesting insight, our experimental design fundamentally cannot measure the phenomena they report.

In our single time-point experimental assay, we measured a cell's mitochondria abundance if it survived TRAIL treatment. Indeed, consistent with the results of Márquez-Jurado et al. [11], our results in Supplemental Figure 11 and Table 1 indicate that the abundance of mitochondria per live cell does not change with TRAIL dosage. If, we were able to measure the mitochondria abundance of the cells that transitioned from live to dead states, perhaps we would have come to the identical conclusion for the HeLa cell experiment. In consequence, we believe that our results and those of Márquez-Jurado et al. [11] complement one another.

Table 5: **Variance Explained.** The variance explained by either $\rho$ or SSC/FSC

| [ABT263] ($\mu M$) | Variance Explained ($\rho$) | Variance Explained (SSC/FSC) |
|---|---|---|
| 0 | 0.0147 | 3.7524 x $10^{-12}$ |
| 1 | 0.0330 | 0.0555 |
| 10 | 0.0259 | 0.1011 |

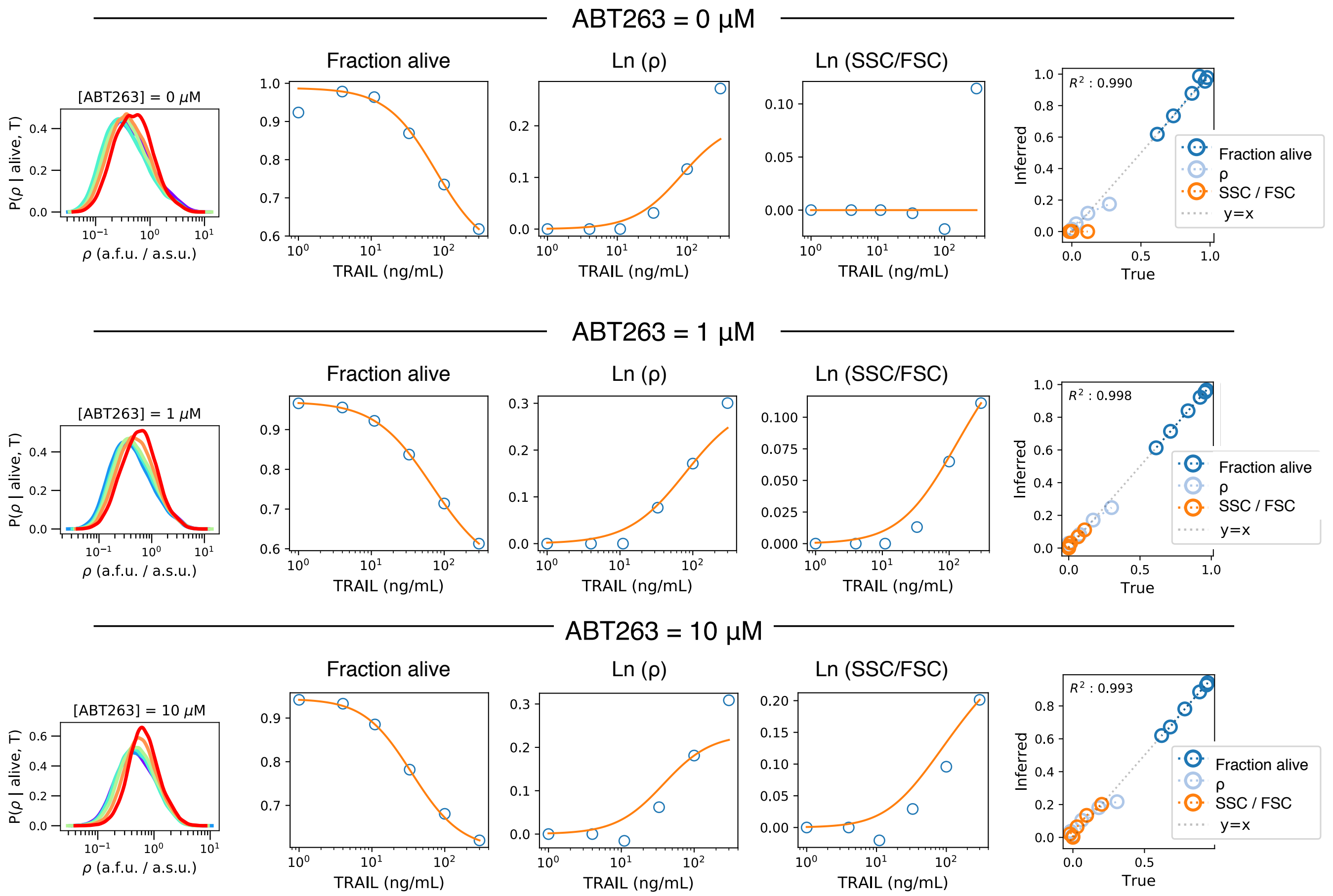


Supplementary Figure 11: **HeLa cell measurements.** Each row displays the identical analysis applied to TRAIL dose response data in which a unique dose of ABT263 has been administered to the cell cultures. The analysis of each row, from left to right, consists of the following. The distribution of Mitotracker DeepRed density among living cells, $P(\text{Ln}(\rho)|\text{alive}, T, ABT263)$. The measurements and model fits of the fraction of cells alive, the average mitochondria density of live cells, and the average side scatter density estimated by the single cell Hill model using the inferred parameters. Lastly, the agreement of the inferred and empirical fraction of cells alive, the average mitochondria and SSC/FSC densities of live cells.

## Supplementary Note 6   Dynamic model of apoptosis

There are several comprehensive dynamic models of the apoptosis pathway in the literature, e.g [2] and [3]. Unique to these studies, our analysis represents a parameter sensitivity analysis of mitochondria surface area and whether it controls regulated cell death. As such, we decided to develop a simpler coarse grained dynamic model of apoptosis where only the biological constituents that are experimentally measured or perturbed are included. In particular we assume that Bax and Bak have interchangeable roles, but for now on will use just Bax for simplicity.

We construct our simplified description of TRAIL induced mitochondria dependent apoptosis by writing the dynamics of active initiator caspase ($c$) as,

$$\frac{dc}{dt} = \underbrace{\Gamma_{co} c_o}_{\text{Spontaneous production}} + \overbrace{\Gamma_{cc} f(T) c_o c}^{\text{autocatalysis}} + \underbrace{\Gamma_{cz} z c_o}_{\text{Feedback from EC}} - \overbrace{\lambda_c c}^{\text{degradation}} . \tag{24}$$

Eq. 24 contains three sources of active caspase production and one process for degradation. The production fluxes are determined by the spontaneous generation of active IC, the autocatalysis of active IC by active IC and TRAIL induced death receptor complex, and feedback from the effector caspase (EC). We assume that abundance of active IC is reduced by a simple one step process. We model Bax activity by its translocation to the mitochondria surface,

$$\frac{dx_{mito}}{dt} = \Gamma_{xc} c - \lambda_x x_{mito}. \tag{25}$$

Take note that the concentration of Bax associated with the mitochondria is in terms of the total cell volume. Additionally, we assume that the mitochondria absorption of Bax monomers is never limiting. Once on the surface of the mitochondria Bax polymerizes, eventually forming pores in the outer-mitochondria membrane. We model this process by computing the time evolution of each Bax $k$-mer concentration, $x_k$ with $k \in \{2, 3, \ldots\}$, as

$$\frac{dx_k}{dt} = \sum_{k,j,i \in \{\omega_k\}} f_{kij}$$

where $f_{kij}$ represents the equilibrium reaction $k_{x+} x_i x_j - k_{x-} x_k$, and $\{\omega_k\}$ is the set of triplets describing all the equilibrium reactions associated with $x_k$. For example,

$$f_{312} = k_{x+} x_1 x_2 - k_{x-} x_3 \qquad \text{and} \qquad f_{211} = k_{x+} x_1 x_1 - k_{x-} x_2.$$

Similarly, we describe the dynamics of Bcl-2 monomers on the mitochondria surface,

$$\frac{db}{dt} = k_{b-} x_b - k_{b+} x_1 b$$

and lastly the activation of effector caspase ($z$),

$$\frac{dz}{dt} = \Gamma_{zx} \frac{N_m}{\Omega} - \lambda_z z,$$

whose production is proportional to the density of Bax pores $N_m$ over the cell volume $\Omega$. Variable definitions can be found in Table 6.

Measurements of both IC and EC activity represents the activation of biomolecules preceding and proceeding mitochondria outer-membrane permeabilization (MOMP, Figure 5A in the main text, [1]). These dynamics show that prior to MOMP, the Initiator Caspase Reporter Protein (IC-RP) and not the Effector Caspase Reporter Protein (EC-RP) increase in abundance [1]. Post-MOMP, EC-RP signal quickly increases and saturates. Such dynamics reflect a system in which the IC-RP levels are changing according to some dynamical equation, while the EC-RP abundance exactly follows.

We employ this intuition to simplify the system of ordinary nonlinear differential equations into a single dynamic equation that represents the concentration of active IC. In doing so, we rely upon the quasi-steady state approximation to eliminate dynamical equations associated with reactions (Table 6) that are much faster than the dynamics of $c$.

We begin our application of the quasi-steady state approximation with the dynamic equation of EC abundance,

$$\frac{dz}{dt} = \Gamma_{zx} \frac{N_m}{\Omega} - \lambda_z z = 0$$
$$\implies z = \frac{N_m}{\alpha_z}, \tag{26}$$

Table 6: Table of variable definitions and assumptions.

| Variable | Definition | Rate assumptions | Constraints |
|---|---|---|---|
| $c$ | Active IC | rate limiting | Mass conservation |
| $c_o$ | Inactive IC | NA | Mass conservation |
| $c_T$ | Total IC | time independent | constant |
| $x_1$ | Mitochondrial associated Bax monomer | fast - quasi-steady-state | detailed balance |
| $f_{kij}$ | equilibrium reaction associated with $k$-mer formation | | |
| | from an $i$-mer and $j$-mer | fast - quasi-steady-state | detailed balance |
| $\Omega$ | Cell volume | constant | equivalent for all cells |
| $\Omega_m$ | Mitochondria surface area | constant | varies from cell-to-cell |
| $x_i$ | Bax $i$-mer | fast - quasi-steady-state | detailed balance |
| $N_i$ | for $i \in \{1, 2, \ldots, m\}$ is number of Bax $i$-mers | NA | Mass conservation |
| $x_m$ | Pores on the mitochondria | fast - quasi-steady-state | detailed balance |
| $B$ | Total concentration of Bcl-2 molecules | constant | Mass conservation |
| $b$ | Bcl-2 monomers | fast- quasi-steady-state | detailed balance |
| $x_b$ | Bcl-2:Bax complex | fast - quasi-steady-state | detailed balance |
| $z$ | Active EC | fast- quasi-steady-state | $Z_T \gg x_m$ |

where $\alpha_z$ is an an effective meta parameter. Moreover, we assumed that the total number of EC is not limiting, specifically that the total number of effector caspase is much more abundant than the number of Bax pores, $N_m$. Incorporating this into Eq. 24,

$$\frac{dc}{dt} = (c_T - c)\left(\Gamma_{co} + \Gamma_{cc} c f(T) - \frac{\Gamma_{cz}}{\alpha_z} N_m\right) - \lambda_c c \tag{27}$$

where we applied IC mass conservation to substitute $c_o = c_T - c$. Here, we see that the feedback from EC is proportional to the number of Bax pores on the mitochondria surface.

Physically, the formation of a Bax pore is simply making an $m$ length polymer from chemical equilibrium reactions that are much faster than the dynamics of $c$. This means that we can apply detailed balance, $f_{kij} = 0$, to all physically possible triplets, $kij$, and consequently simplify our system of ODE's to algebraic equations,

$$f_{k(k-1)1} = k_{x+} x_{k-1} x_1 - k_{x-} x_k = 0 \qquad \text{for } k \in \{2, 3, \ldots, m\}$$
$$\implies x_k = k_x \left(\frac{x_1}{k_x}\right)^k,$$

where $k_x = k_{x-}/k_{x+}$. From this operation, we are able to compute the concentration of any $k$-mer from knowledge of the concentration of Bax monomers alone. Similar to the dynamics of Bax $k$-mer formation are the dynamics of Bcl-2:Bax dimers,

$$\frac{dx_b}{dt} = 0 \qquad \implies \qquad b = \frac{k_b x_b}{x_1} \tag{28}$$

and incorporating into the Bcl-2 mass conservation equation, $B = x_b + b$,

$$x_b = \frac{B x_1}{k_b + x_1}.$$

We incorporate the preceding equations by means of the mass conservation equation of Bax,

$$\begin{aligned} N_{\text{Bax Total}} &= N_{\text{Bcl2:Bax}} + \sum_{k=1}^{m} k N_{k\text{-mer}} \\ &= \Omega_m x_b + \Omega_m k_x \sum_{k=1}^{m} k \left(\frac{x_1}{k_x}\right)^k \\ &= \frac{N_B N_1}{k_b \Omega_m + N_1} + \Omega_m k_x \sum_{k=1}^{m} k \left(\frac{N_1}{\Omega_m k_x}\right)^k . \end{aligned} \tag{29}$$

where $\Omega_m$ is the surface area of mitochondria. In Eq. 29 we see that by increasing the mitochondria surface area, the propensity to form Bax $k$-mers and Bcl-2:Bax dimers decreases.

While we don't immediately know the total number of Bax monomers on the mitochondria surface, $N_{\text{Bax Total}}$, we do know the concentration of total active Bax, $x_{mito}$. From Eq. 25, we see that $x_{mito}$ is strictly dependent on the concentration of IC,

$$\frac{dx_{mito}}{dt} = \Gamma_{xc} c - \lambda_x x_{mito} = 0 \qquad \implies \qquad x_{mito} = \frac{c}{\alpha_x},$$

where $\alpha_x$ is the ratio of the degradation to production rates. Then substituting into Eq. 29,

$$\Omega \frac{c}{k_x \alpha_x} = \frac{1}{k_x} \frac{N_B N_1}{k_b \Omega_m + N_1} + \Omega_m \sum_{k=1}^{m} k \left(\frac{N_1}{k_x \Omega_m}\right)^k . \tag{30}$$

Eq. 30 has only a single root which is not analytically accessible. Only a single root exists, because all coefficients and variables in the equation are restricted to positive numbers. Consequently, numerical solutions for $x_1$ are unique and physical.

Incorporating these findings in Eq. 27 we have a simplified description of active IC dynamics,

$$\frac{dc}{dt} = (c_T - c)\left(\Gamma_{co} + \Gamma_{cc} f(T) c + \frac{\Omega_m k_x \Gamma_{cz}}{\alpha_z}\left(\frac{N_1}{k_x \Omega_m}\right)^m\right) - \lambda_c c. \tag{31}$$

Eqs. 30, 31 include all the information of our coarse-grained description. However, in its current form, the model is in terms of specific units, e.g. concentration, which are not available in our measurements. To be able to map our model results to data, we transformed Eqs. 30, 31 into non-dimensional descriptions.

Let the dimensionless time $\tau = \lambda_c t$, IC concentration $y = \frac{c}{k_x}$, and Bax monomer concentration $\zeta = \frac{N_1}{k_x}$ then Eq. 31 can be re-written as,

$$\frac{dy}{d\tau} = (y_T - y)\left(r + \gamma f(T)y + \Omega_m \mu \left(\frac{\zeta}{\Omega_m}\right)^m\right) - y \tag{32}$$

where $\zeta$ is the solution to,

$$\frac{y}{\alpha} = \frac{\beta\zeta}{\kappa_d \Omega_m + \zeta} + \Omega_m \sum_{k=1}^{m} k \left(\frac{\zeta}{\Omega_m}\right)^k. \tag{33}$$

The coarse-grained variable definitions can be found in Table 7 and parameters in Table 8.

## Supplementary Note 6.1 Incorporating Bcl-2 inhibition

The ability of Bcl-2 to sequester Bax monomers can be reduced by the application of a small molecule inhibitor of Bcl-2 activity, e.g. ABT263. Specifically, its action decreases the number of Bcl-2:Bax complexes that can form, and consequently is incorporated into our model by means of Bax mass conservation, Eq. 29. To derive the equation of Bcl-2:Bax complexes in terms of the inhibitor we must begin with the steady state chemical equation,

$$\frac{db_I}{dt} = k_+^I bI - k_- b_I = 0, \qquad \Longrightarrow \qquad b_I = \frac{bI}{k_I}. \tag{34}$$

Then by mass conservation of Bcl-2,

$$N_B = N_{\text{Bcl-2 monomers}} + N_{\text{Bcl-2:Bax}} + N_{\text{Bcl-2:I}}. \tag{35}$$

and substituting Eqs. 28,34 into Eq. 35,

$$\begin{aligned} N_B &= \Omega_m \frac{k_b N_{\text{Bcl-2:Bax}}}{N_1} + N_{\text{Bcl-2:Bax}} + \Omega_m \frac{bI}{k_I} \\ &= N_{\text{Bcl-2:Bax}} \left[1 + \frac{k_b \Omega_m}{N_1}\left(1 + \frac{I}{k_I}\right)\right] \end{aligned}$$

in which we see that,

$$N_{\text{Bcl-2:Bax}} = \frac{N_B N_1}{\Omega_m k_b \left(1 + \frac{I}{k_I}\right) + N_1}.$$

Which we can replace the dimensionless equation of constraint (Eq. 33) with,

$$\frac{y}{\alpha} = \frac{\beta\zeta}{\Omega_m \kappa_d (1 + \frac{I}{k_I}) + \zeta} + \Omega_m \sum_{k}^{m} k \left(\frac{\zeta}{\Omega_m}\right)^k.$$

## Supplementary Note 6.2 Complete model

We incorporate mitochondria abundance, Bcl-2 family proteins, and Bcl-2 inhibitors to the dimensionless Eq. 32 to arrive at our mathematical description of apoptosis,

$$\frac{dy}{d\tau} = (y_T - y)\left(\overbrace{r}^{\text{Spontaneous production}} + \underbrace{\gamma y \frac{T^{n_T}}{T^{n_T} + \text{EC}_{50}^{n_T}}}_{\text{Receptor activation}} + \overbrace{\Omega_m \mu \left(\frac{\zeta}{\Omega_m}\right)^m}^{\text{EC Feedback}}\right) - y. \tag{36}$$

Table 7: Coarse-grained variables

| Variable | Description | units |
|---|---|---|
| $y$ | active form of Initiator Caspase | dimensionless |
| $\zeta$ | Bax monomers on the mitochondria surface | dimensionless |
| $\beta$ | total Bcl-2 molecules on mitochondria surface | dimensionless |
| $I$ | concentration of Bcl-2 inhibitor ABT263 | nM |
| $T$ | TRAIL dose | ng/mL |

These dynamics are constrained such that the total number of Bax and Bcl-2 molecules are conserved,

$$\frac{y}{\alpha} = \overbrace{\frac{\zeta\beta}{\Omega_m \kappa_d (1 + \frac{I}{k_I}) + \zeta}}^{\text{Bax-Bcl2 Complex}} + \underbrace{\Omega_m \sum_k^m k \left(\frac{\zeta}{\Omega_m}\right)^k}_{\text{Bax } k-\text{mers}} .. \tag{37}$$

To simulate dynamics of Eq. 36 we used the fourth-order Runge-Kutta algorithm, where in addition to computing the fluxes at each time step we numerically solve Eq. 37 for $\zeta$ using Scipy's optimize.bisect function. A detailed list of variable (Table 7) and parameter definitions (Table 8) are provided.

In addition to simulating the dynamics of $y$, we use Eqs. 36, 37 to study the bifurcation diagrams of the apoptosis pathway and to infer the rate constants $r, \mu$ and $\gamma$ from single cell temporal measurements of IC. The bifurcation diagrams in Figures 5D-G of the main text, were constructed by numerically solving for the fixed points of Eq. 36 with Scipy's optimize.bisect function for different mitochondria surface areas. Details on the methods to infer dynamic parameters can be found in the section on quantification and statistical analysis.

## Supplementary Note 6.3 Stochastic dynamic simulations

We simulated the stochastic dynamics of active initiator caspase abundance using the chemical Langevin equation. We derived the Langevin equation by approximating the chemical Master equation to second order, the so-called Fokker-Planck equation [14]. The first order "drift" and second "diffusion" terms represent the deterministic reactions and the magnitude of the stochastic force in the Langevin equation. This representation is an approximate model to the inherent stochasticity of chemical reactions.

Consider the Master equation corresponding to the activation and degradation of initiator caspase molecules,

$$\frac{\partial P_n(t)}{\partial t} = g(n-1)P_{n-1}(t) + d(n+1)P_{n+1}(t) - (g(n) + d(n))P_n(t) \tag{38}$$

where $n$ represents the number of molecules, while $g(n)$ and $d(n)$ are functions representing the generative and degradation transition probabilities, respectively. Instead of working with the discrete variable $n$, we may re-express Eq. 38 in terms of our dimensionless density variable $y = \frac{n}{k_x\Omega}$ and jump sizes $\ell = \frac{1}{k_x\Omega}$, with $\Omega$. Similar to Kepler and Elston [10], let,

$$P_n(t) = \int_{y=n-\ell/2}^{n+\ell/2} P(y,t)dy$$

and substitute,

$$g(n) = k_x\Omega f_+(y), \qquad \text{and} \qquad d(n) = k_x\Omega f_-(y)$$

into the Eq. 38,

$$\frac{\partial P(y,\tau)}{\partial \tau} = k_x\Omega\left[f_+(y-\ell)P(y-\ell,\tau) + f_-(y+\ell)P(y+\ell,\tau) - (f_+(y) + f_-(y))\,P(y,\tau)\right], \tag{39}$$

the chemical Master equation in terms of $y$, $\tau$, and $\ell$ [8]. Next, we use this chemical Master equation to derive the corresponding Fokker-Planck equation.

Lets write Eq. 39 in terms of $f(y)$ and $P(y,\tau)$ by applying the shift operator [14,15],

$$f_j(y \pm \ell)P(y \pm \ell) = e^{\pm \ell \partial/\partial y} f_j(y)P(y,\tau)$$

Table 8: Simulation parameters

| Parameter | Description | Units | Value | Jurkat | MDA-MB-231 |
|---|---|---|---|---|---|
| $r$ | spontaneous production rate of $y$ | dimensionless | - | 0.025 | 0.014 |
| $\mu$ | feedback strength from the Effector Caspase | dimensionless | - | 137.667 | 257.808 |
| $\gamma$ | autocatalysis feedback strength of Initiator Caspase | dimensionless | - | 3.468 | 5.336 |
| $\kappa_d$ | the effective binding affinity of Bcl-2 with Bax | dimensionless | - | 2.5 | 0.1 |
| $y_T$ | total abundace of Initiator Caspase | dimensionless | 0.865 | - | - |
| $m$ | the average number of bax monomers per pore | dimensionless | 5 | - | - |
| $n_T$ | Hill coefficient of TRAIL recptor activation by TRAIL | 2 | - | - | |
| $\alpha$ | Convert $y$ to mitochondria localized Bax | dimensionless | 0.2 | - | - |
| $\beta$ | total abundance of Bcl-2 | dimensionless | 1 | - | - |
| $k_I$ | binding affinity of ABT263 with Bcl-2 | nM | 1 | - | - |
| $\Omega_m$ | Mitochondria surface area | dimensionless | $k_x\Omega_m$=0.05 | - | - |

with $j \in \{+,-\}$,

$$\frac{\partial P(y,\tau)}{\partial \tau} = k_x\Omega \left[\left(e^{-\ell\partial/\partial y} - 1\right) f_+(y) + \left(e^{\ell\partial/\partial y} - 1\right) f_-(y)\right] P(y,\tau). \tag{40}$$

If we expand Eq. 40 in terms of $\ell$ about $\ell = 0$ we obtain the infinite series,

$$\frac{\partial P(y,\tau)}{\partial \tau} = \sum_{n=0}^{\infty} k_x\Omega \frac{\ell^n}{n!}\frac{\partial^n}{\partial y^n}\left[(-1)^n f_+(y) + f_-(y)\right] P(y,\tau), \tag{41}$$

which at first glance appears needlessly complex. However, we may statistically interpret the coefficients of Eq. 41 through the perspective of the Kramers-Moyal expansion,

$$\frac{\partial P(y,\tau)}{\partial \tau} = \sum_{n=1}^{\infty} \frac{\partial^n}{\partial y^n} D^{(n)}(y) P(y,\tau), \tag{42}$$

where the coefficients $D^{(n)}(y) = \lim_{\Delta\tau\to 0} \frac{\ell^n}{n!}\frac{1}{\Delta\tau} M_n(y,\tau+\Delta\tau,\tau)$ [14,15] represent the change of the $n^{th}$ moment in the infinitesimal time $\Delta\tau$. Under this interpretation, the zeroth order coefficient of Eq. 41 must be zero due to the fact that probability densities always normalize. Meanwhile, the first and second order coefficients represent the drift and diffusion of the stochastic process governing $y$. Truncating Eq. 41 at second order manifests as the so-called Fokker-Planck equation of our dimensionless chemical system, with drift and diffusion described by,

$$D^{(1)}(y) = f_+(y) - f_-(y) \tag{43}$$

$$D^{(2)}(y) = \frac{1}{2k_x\Omega}\left(f_+(y) + f_-(y)\right). \tag{44}$$

Then the corresponding chemical Langevin equation is simply,

$$\frac{dy}{d\tau} = D^{(1)}(y) + \sqrt{D^{(2)}(y)}\xi(t) \tag{45}$$

where,

$$\langle \xi(t) \rangle = 0 \qquad \text{and} \qquad \langle \xi(t)\xi(t+\tau)\rangle = \delta(\tau),$$

which is in agreement with derivations [8]. We simulate the dynamics of initiator caspase abundance, $y$, using the Itô interpretation of the Langevin equation in Eq. 45.

## Supplementary Note 6.4 Inferring dynamic model parameters

To constrain cell type specific parameter sets for our dynamic model of apoptosis we corroborated the predicted dynamics with previously published data as the best proxy available given there are no measurements for the dynamics of Initiator and effector caspases in the cell lines we used. Specifically, we fit the parameters $r$, $\gamma$, and $\mu$ to temporal single cell measurements of a reporter of initiator caspase activity (IC-RP) published in Albeck et al. 2008.

To fit the data to our dimensionless model, we transformed time and the IC-RP measurements to dimensionless units. First, we aligned the trajectories of ten individual cells by the time in which MOMP occurred, and restricted the time interval of interest, $t$, to $t = t_{\text{MOMP}} \pm$ 3.3 hours. We then introduced dimensionless time as $\tau = t/\langle t\rangle$. The IC-RP signal was normalized so that the signal ranges between 0 and 1. Specifically, the normalized signal, $y$, from the $i^{th}$ time point measurement of the $j^{th}$ cell was computed by,

$$y_{ij} = \frac{\text{IC-RP}_{ij} - \min(\text{IC-RP}_j)}{\max(\text{IC-RP}_j) - \min(\text{IC-RP}_j)}.$$

We infer the model parameters by minimizing the negative log-likelihood of the model evaluated at each timepoint under saturating ($s = 1$)and zero ($s = 0$) TRAIL stimulation,

$$\hat{\boldsymbol{\theta}} = \underset{\boldsymbol{\theta}}{\operatorname{argmin}} \left\{ \sum_{i=1}^{N} \frac{(\langle y_i\rangle - f(\tau_i; s=1,\boldsymbol{\theta}))^2}{\sigma_i^2} + \frac{(\langle y_0\rangle - f(\tau_i; s=0,\boldsymbol{\theta}))^2}{\langle y_0\rangle} \right\}. \tag{46}$$

Here, $f(\tau_i; s,\boldsymbol{\theta})$ is the signal predicted by the deterministic dynamic model evaluated at time $\tau_i$ and stimulation strength $s$. The $\langle y_i\rangle$ and $\sigma_i^2$ are the mean and the standard error of the mean of the normalized experimental data

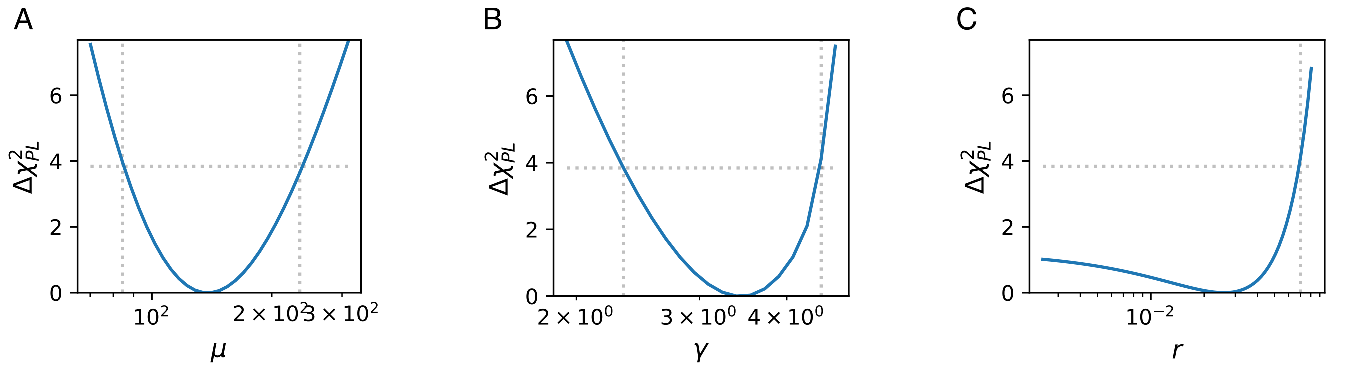


Supplementary Figure 12: **Profile likelihood of Jurkat Model.**

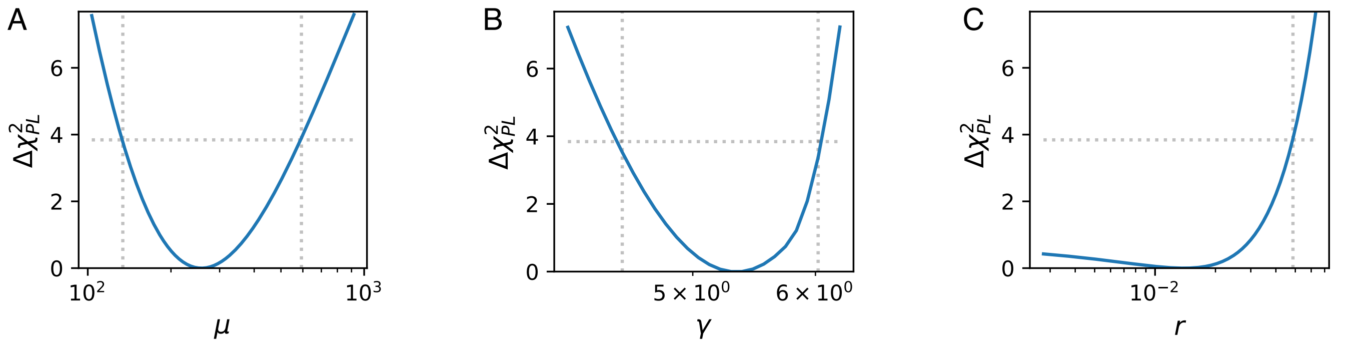


Supplementary Figure 13: **Profile likelihood of MDA-MB-231 Model.**

measured at time point $\tau_i$. Lastly, for consistency with our model, we require that all inferred model parameters to be positive values. The optimization in Eq. 46 was performed using Scipy's nonlinear optimization tool, optimize.fmin, [9].

Often, estimating the confidence intervals of the inferred parameters from biological nonlinear dynamic models is challenging. This is on account, that the negative log likelihood of a parameter set may not be that of a multivariate Normal distribution. Indeed, practioners have long appreciated that such models can exhibit irregular log-likelihood functions in the form of many local minima, structural non-identifiability or practical non-identifiability [13]. One way to analyze the extent of the log-likelihood function irregularities is to estimate the confidence intervals from the profile likelihood. The profile likelihood of the $i^{th}$ parameter is the the error Eq. 46, or $\chi^2$, estimated by optimizing all parameters $\theta_{j\neq i}$ given specific values of $\theta_i$. By plotting the profile likelihood for each parameter we can graphically analyze the models goodness-of-fit and estimate the corresponding confidence intervals.

In Figures 12, 13 we show the profile likelihood of each parameter in our Jurkat and MDA-MB-231 models, respectively. For each parameter we estimated the $\Delta\chi^2 = \chi^2(\theta_i) - \min(\chi^2(\theta_i)$ for 100 values logarithmically spaced on the interval $[\hat{\theta}_i/10,\ 100\hat{\theta}_i]$. We then computed the 95% confidence intervals by finding $\theta$ such that the profile likelihood and the $\Delta\chi^2$, computed for 95% quantile scipy.stats.chi2 [9], intersect. Although the profile likelihood for $r$ is practically non-identifiable as very shallow and unbounded below, given that for the biological interpretation of the model we were only interested in positive values, the origin was considered as a lower bound.

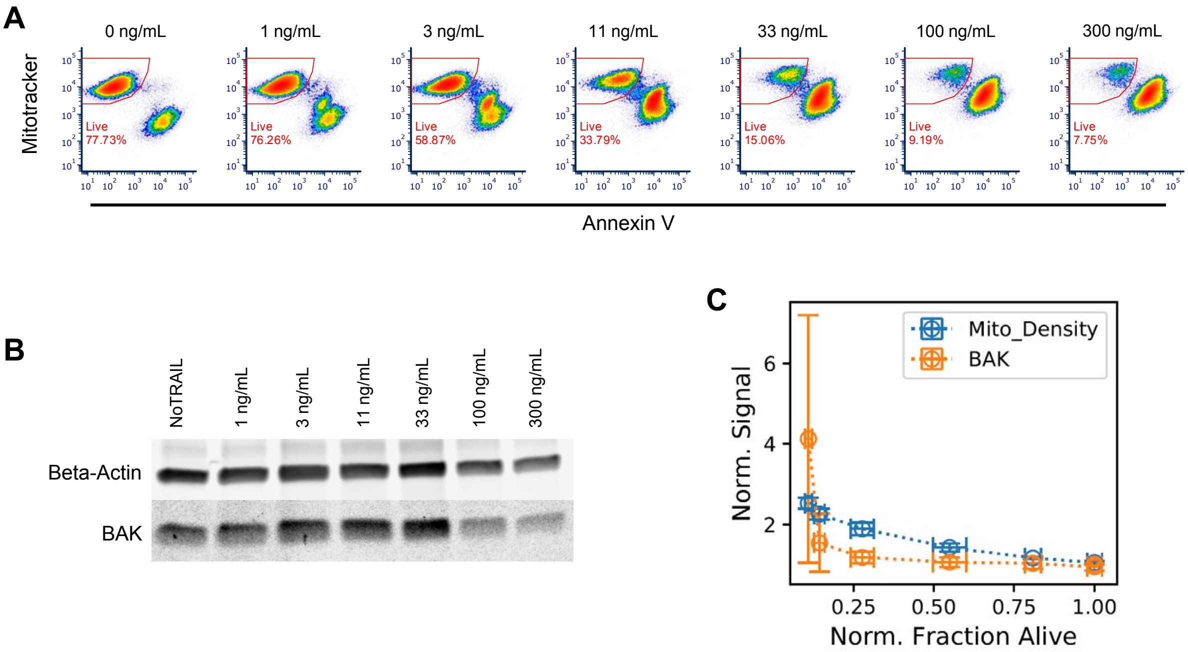


Supplementary Figure 14: **Mitochondria density and Bak measurements in surviving Jurkat cells.** **A.** Flow cytometry 2D plots of 7-point dose response to TRAIL at 4h. Live cell gate with fraction of cells that survived TRAIL shown on plots. Gating strategy as described in Sup.Note 1. Measurements (a.f.u.) of Mitotracker DeepRed represented on the yy axes. Measurements (a.f.u.) of Annexin V-FITC represented on the xx axes. Plots shown representative of three replicates. **B.** Western immunoblot of Bak and Actin in whole cell lysates harvested from the surviving cells isolated by FACS sorter from the "live gate" depicted in **A**. Blot shown representative of three replicates. **C.** Levels of Bak protein and Mitochondria density measured by western and flow cytometry, respectively, in surviving cells for each dose of TRAIL. For each sample, signal intensity of Bak bands from three blots were divided by corresponding intensity of actin bands, then normalized to the sample without TRAIL. Signal for Mitochondria density in surviving cells from the "live gate" was computed by dividing the average MitoTracker fluorescence intensity (a.f.u.) by the average forward scatter values (a.s.u.), and then normalized to the sample without TRAIL. Normalized signals of Bak protein and Mitochondria density were plotted against average fraction alive, in orange and blue, respectively. Error bars represent three replicates. Antibodies: beta-Actin (clone D6A8, Cell Signaling, cat. No. 8457); Bak (clone D4E4, Cell Signaling, cat. No. 12105);

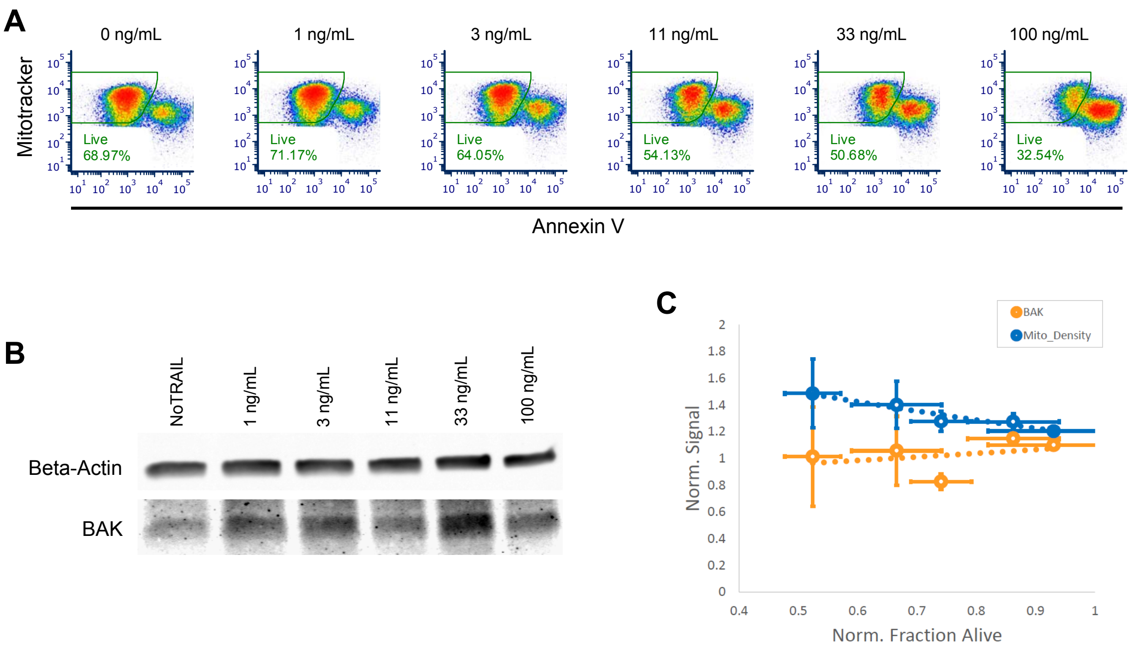


Supplementary Figure 15: **Mitochondria density and Bak measurements in surviving MDA-MB-231 cells.** **A.** Flow cytometry 2D plots of 6-point dose response to TRAIL and 1$\mu$M ABT263 for all doses at 4h. Live cell gate with fraction of cells that survived TRAIL shown on plots. Gating strategy as described in Sup.Note 1. Measurements (a.f.u.) of Mitotracker DeepRed represented on the yy axes. Measurements (a.f.u.) of Annexin V-FITC represented on the xx axes. Plots shown representative of three replicates. **B.** Western immunoblot of Bak and Actin in whole cell lysates harvested from the surviving cells isolated by FACS sorter from the "live gate" depicted in **A**. Blot shown representative of three replicates. **C.** Levels of Bak protein and Mitochondria density measured by western and flow cytometry, respectively, in surviving cells for each dose of TRAIL. For each sample, signal intensity of Bak bands from three blots were divided by corresponding intensity of actin bands, then normalized to the sample without TRAIL. Signal for Mitochondria density in surviving cells from the "live gate" was computed by dividing the average MitoTracker fluorescence intensity (a.f.u.) by the average forward scatter values (a.s.u.), and then normalized to the sample without TRAIL. Normalized signals of Bak protein and Mitochondria density were plotted against average fraction alive, in orange and blue, respectively. Error bars represent two replicates. Antibodies: beta-Actin (clone D6A8, Cell Signaling, cat. No. 8457); Bak (clone D4E4, Cell Signaling, cat. No. 12105);

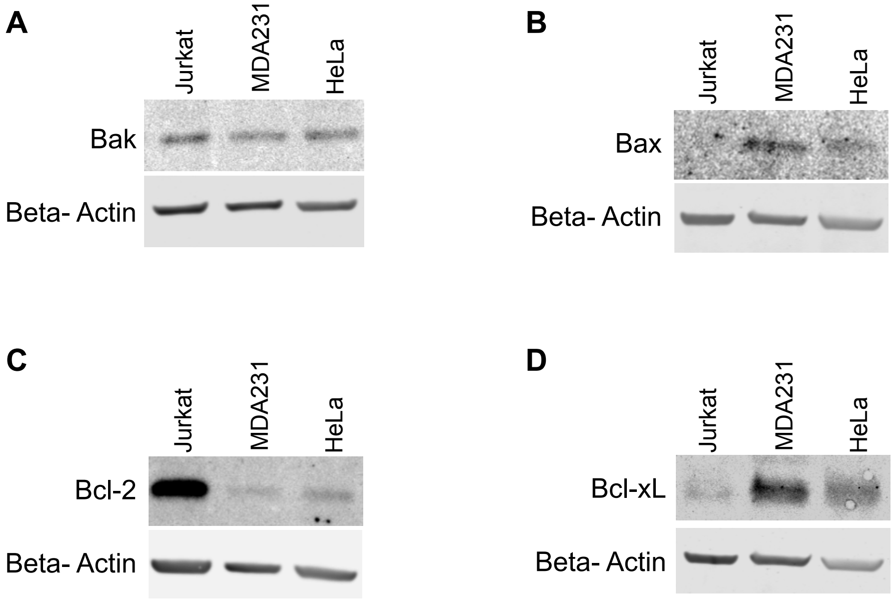


Supplementary Figure 16: **Bcl-2-family proteins in Jurkat, MDA-MB-231 and Hela cells.**Representative western blot results showing relative abundances of Bcl-2-family proteins in different cell lines. Briefly, whole cell lysates were harvested from Jurkat, MDA231, and HeLa cells as previously described [5]. Total protein (50 $\mu$g per well) was loaded into PAGE gels (cat. No. 456-9034), ran for 180 minutes (50 V), and then transferred to nitrocellulose membranes (0.45$\mu$m, Bio-Rad, cat. No. 1620145) for 70 minutes (220 mA). Membranes were blocked and probed with antibodies against Actin for all blots, **A.**Bak **B.** Bak **C.** Bcl-2 **D.** Bcl-XL. Antibodies: beta-Actin (clone D6A8, Cell Signaling, cat. No. 8457); Bak (clone D4E4, Cell Signaling, cat. No. 12105); Bax (clone 2D2, Santa Cruz, cat. No. 20067); Bcl-2 (clone 100, Santa Cruz, cat. No. 509); Bcl-XL (clone H-5, Santa Cruz, cat. No. 8392).

## Supplementary References


1. John G. Albeck, John M. Burke, Bree B. Aldridge, Mingsheng Zhang, Douglas A. Lauffenburger, and Peter K. Sorger. Quantitative analysis of pathways controlling extrinsic apoptosis in single cells. *Molecular Cell*, 30(1):11 – 25, 2008.

2. John G Albeck, John M Burke, Sabrina L Spencer, Douglas A Lauffenburger, and Peter K Sorger. Modeling a snap-action, variable-delay switch controlling extrinsic cell death. *PLoS Biol*, 6(12):e299, 12 2008.

3. Mehdi Bouhaddou, Anne Marie Barrette, Rick J Koch, Matthew S DiStefano, Eric A Riesel, Alan D Stern, Luis C Santos, Annie Tan, Alex Mertz, and Marc R Birtwistle. An integrated mechanistic model of pan-cancer driver pathways predicts stochastic proliferation and death. *bioRxiv*, 2017.

4. Long Cai, Nir Friedman, and X Sunney Xie. Stochastic protein expression in individual cells at the single molecule level. *Nature*, 440(7082):358, 2006.

5. Andrea Degasperi, Marc R Birtwistle, Natalia Volinsky, Jens Rauch, Walter Kolch, and Boris N Kholodenko. Evaluating strategies to normalise biological replicates of western blot data. *PloS one*, 9(1):e87293, 2014.

6. Michael B Elowitz, Arnold J Levine, Eric D Siggia, and Peter S Swain. Stochastic gene expression in a single cell. *Science*, 297(5584):1183–1186, 2002.

7. Nir Friedman, Long Cai, and X Sunney Xie. Linking stochastic dynamics to population distribution: an analytical framework of gene expression. *Physical review letters*, 97(16):168302, 2006.

8. Daniel T. Gillespie. The chemical langevin equation. *The Journal of Chemical Physics*, 113(1):297–306, 2000.

9. Eric Jones, Travis Oliphant, Pearu Peterson, et al. SciPy: Open source scientific tools for Python, 2001–.

10. Thomas B Kepler and Timothy C Elston. Stochasticity in transcriptional regulation: origins, consequences, and mathematical representations. *Biophysical journal*, 81(6):3116–3136, 2001.

11. Silvia Márquez-Jurado, Juan Díaz-Colunga, Ricardo Pires Neves, Antonio Martinez-Lorente, Fernando Almazán, Raúl Guantes, and Francisco J Iborra. Mitochondrial levels determine variability in cell death by modulating apoptotic gene expression. *Nature communications*, 9(1):389, 2018.

12. Robert J. Prill, Robert Vogel, Guillermo A. Cecchi, Grégoire Altan-Bonnet, and Gustavo Stolovitzky. Noise-driven causal inference in biomolecular networks. *PLoS ONE*, 10(6):e0125777, 06 2015.

13. A. Raue, C. Kreutz, T. Maiwald, J. Bachmann, M. Schilling, U. Klingm uller, and J. Timmer. Structural and practical identifiability analysis of partially observed dynamical models by exploiting the profile likelihood. *Bioinformatics*, 25(15):1923–1929, 2009.

14. Hannes Risken. *The Fokker-Planck Equation.* Springer, 1996.

15. N G Van Kampen. *Stochastic Processes in Physics and Chemistry.* Elsevier, North-Holland, Amsterdam, 1992.
