# Supplementary material for: Mitochondrial origins of fractional control in regulated cell death
Source: Nat Commun. 2019 Mar 21;10:1313. doi: 10.1038/s41467-019-09275-x (PMC6428895; doi:10.1038/s41467-019-09275-x)
Supplement: Supplementary file 3 — Reporting Summary [file 41467_2019_9275_MOESM3_ESM.pdf]

nature research

Corresponding author(s): Pablo Meyer

Last updated by author(s): 02/26/2019

Reporting Summary

Nature Research wishes to improve the reproducibility of the work that we publish. This form provides structure for consistency and transparency in reporting. For further information on Nature Research policies, see Authors & Referees and the Editorial Policy Checklist.

Statistics

For all statistical analyses, confirm that the following items are present in the figure legend, table legend, main text, or Methods section.

n/a	Confirmed	
☐	☒	The exact sample size (*n*) for each experimental group/condition, given as a discrete number and unit of measurement
☐	☒	A statement on whether measurements were taken from distinct samples or whether the same sample was measured repeatedly
☐	☒	The statistical test(s) used AND whether they are one- or two-sided<br>*Only common tests should be described solely by name; describe more complex techniques in the Methods section.*
☐	☒	A description of all covariates tested
☐	☒	A description of any assumptions or corrections, such as tests of normality and adjustment for multiple comparisons
☐	☒	A full description of the statistical parameters including central tendency (e.g. means) or other basic estimates (e.g. regression coefficient) AND variation (e.g. standard deviation) or associated estimates of uncertainty (e.g. confidence intervals)
☐	☒	For null hypothesis testing, the test statistic (e.g. *F*, *t*, *r*) with confidence intervals, effect sizes, degrees of freedom and *P* value noted<br>*Give P values as exact values whenever suitable.*
☒	☐	For Bayesian analysis, information on the choice of priors and Markov chain Monte Carlo settings
☒	☐	For hierarchical and complex designs, identification of the appropriate level for tests and full reporting of outcomes
☒	☐	Estimates of effect sizes (e.g. Cohen's *d*, Pearson's *r*), indicating how they were calculated

Our web collection on statistics for biologists contains articles on many of the points above.

Software and code

Policy information about availability of computer code

Data collection	All programs and their used are detailed in the manuscript
Data analysis	All code for analysis is available in the page github.com/depictive

For manuscripts utilizing custom algorithms or software that are central to the research but not yet described in published literature, software must be made available to editors/reviewers. We strongly encourage code deposition in a community repository (e.g. GitHub). See the Nature Research guidelines for submitting code & software for further information.

Data

Policy information about availability of data

All manuscripts must include a data availability statement. This statement should provide the following information, where applicable:

- Accession codes, unique identifiers, or web links for publicly available datasets
- A list of figures that have associated raw data
- A description of any restrictions on data availability

Accession codes, unique identifiers, and web links for publicly available datasets were provided as their association to the figures

Field-specific reporting

Please select the one below that is the best fit for your research. If you are not sure, read the appropriate sections before making your selection.

☒ Life sciences ☐ Behavioural & social sciences ☐ Ecological, evolutionary & environmental sciences

For a reference copy of the document with all sections, see nature.com/documents/nr-reporting-summary-flat.pdf

Life sciences study design

All studies must disclose on these points even when the disclosure is negative.

Sample size	The number of cells were chosen in order to perform a standard FACS assay
Data exclusions	no data were excluded from the analysis
Replication	Biological and technical replicates of all experiments were performed
Randomization	this is not relevant as proper controls were used
Blinding	this is not relevant as proper controls were used

Reporting for specific materials, systems and methods

We require information from authors about some types of materials, experimental systems and methods used in many studies. Here, indicate whether each material, system or method listed is relevant to your study. If you are not sure if a list item applies to your research, read the appropriate section before selecting a response.

Materials & experimental systems

n/a	Involved in the study	
☐	☒	Antibodies
☐	☒	Eukaryotic cell lines
☒	☐	Palaeontology
☒	☐	Animals and other organisms
☒	☐	Human research participants
☒	☐	Clinical data

Methods

n/a	Involved in the study	
☒	☐	ChIP-seq
☐	☒	Flow cytometry
☒	☐	MRI-based neuroimaging

Antibodies

Antibodies used	beta-Actin (clone D6A8, Cell Signaling, cat. No. 8457); Bak (clone D4E4, Cell Signaling, cat. No. 12105); Bax (clone 2D2, Santa Cruz, cat. No. 20067); Bcl-2 (clone 100, Santa Cruz, cat. No. 509); Bcl-XL (clone H-5, Santa Cruz, cat. No. 8392).
Validation	cells with knockdowns for BAK and BAX were used as controls

Eukaryotic cell lines

Policy information about cell lines

Cell line source(s)	Jurkat E6-1 cells from ATCC (TIP-152), MDA-MB-231 cells from ATCC (no.HTB-26) and HeLa cells (ATCC CCL2) were were purchased from ATCC
Authentication	standard procedure from ATCC
Mycoplasma contamination	mycoplasma contamination was performed using standard procedures
Commonly misidentified lines (See ICLAC register)	NA

Flow Cytometry

Plots

Confirm that:

☒ The axis labels state the marker and fluorochrome used (e.g. CD4-FITC).

☒ The axis scales are clearly visible. Include numbers along axes only for bottom left plot of group (a 'group' is an analysis of identical markers).

☒ All plots are contour plots with outliers or pseudocolor plots.

☒ A numerical value for number of cells or percentage (with statistics) is provided.

Methodology

Sample preparation	cells were pelleted by centrifugation for 5 minutes at 100 x g, and then resuspended in 1x PBS and stained with 200 nM MitoTracker Deep Red (Life Technologies, cat. M22426) for 10 minutes at 37C. MitoTracker staining was quenched with full cell culture medium, followed by centrifugation for 5 minutes at 100 x g. Cells were resuspended in cell culture media at a density of 1 x 10^{6} per mL, in which 100000 were transferred to each experimental well of a flat-bottom 96-well plate. Cells were then incubated at 37C for 4 hours with different doses of Superkiller TRAIL (Enzo Life Sciences cat. ALX-201-115) and/or ABT263 (ApexBio cat. A3007). After drug treatment, cells were transferred to a v-bottom 96-well plate, pelleted by centrifugation at 1,000 x g, stained with FITC-conjugated Annexin V (Biolegend cat. 640945), and then measured by flow cytometry.
Instrument	Flow cytometry measurements were conducted on a BD LSRII
Software	FCS express 6 from De Novo Software and BD FACSdiva
Cell population abundance	100 000 cells
Gating strategy	Elimination of debris measurements identified as FSC-A low and SSC-A low. Elimination of doublet measurements by gating along the region FSC-A = FSC-H. Gate on cells that were successfully stained by Mitotracker Deep Red. Live cells identified as Annexin V negative.

☒ Tick this box to confirm that a figure exemplifying the gating strategy is provided in the Supplementary Information.
